# New development of atomic layer deposition: processes, methods and applications

**DOI:** 10.1080/14686996.2019.1599694

**Published:** 2019-05-23

**Authors:** Peter Ozaveshe Oviroh, Rokhsareh Akbarzadeh, Dongqing Pan, Rigardt Alfred Maarten Coetzee, Tien-Chien Jen

**Affiliations:** a Mechanical Engineering Science Department, Faculty of Engineering and the Built Environment, University of Johannesburg, Johannesburg, South Africa; b Department of Engineering Technology, University of North Alabama, Florence, AL, USA

**Keywords:** Atomic layer deposition, Thin film, Molecular dynamics, Computational fluid dynamics, 10 Engineering and Structural materials, 102 Porous / Nanoporous / Nanostructured materials, 306 Thin film / Coatings, 400 Modeling / Simulations

## Abstract

Atomic layer deposition (ALD) is an ultra-thin film deposition technique that has found many applications owing to its distinct abilities. They include uniform deposition of conformal films with controllable thickness, even on complex three-dimensional surfaces, and can improve the efficiency of electronic devices. This technology has attracted significant interest both for fundamental understanding how the new functional materials can be synthesized by ALD and for numerous practical applications, particularly in advanced nanopatterning for microelectronics, energy storage systems, desalinations, catalysis and medical fields. This review introduces the progress made in ALD, both for computational and experimental methodologies, and provides an outlook of this emerging technology in comparison with other film deposition methods. It discusses experimental approaches and factors that affect the deposition and presents simulation methods, such as molecular dynamics and computational fluid dynamics, which help determine and predict effective ways to optimize ALD processes, hence enabling the reduction in cost, energy waste and adverse environmental impacts. Specific examples are chosen to illustrate the progress in ALD processes and applications that showed a considerable impact on other technologies.

## Graphical abstract

**Figure:**
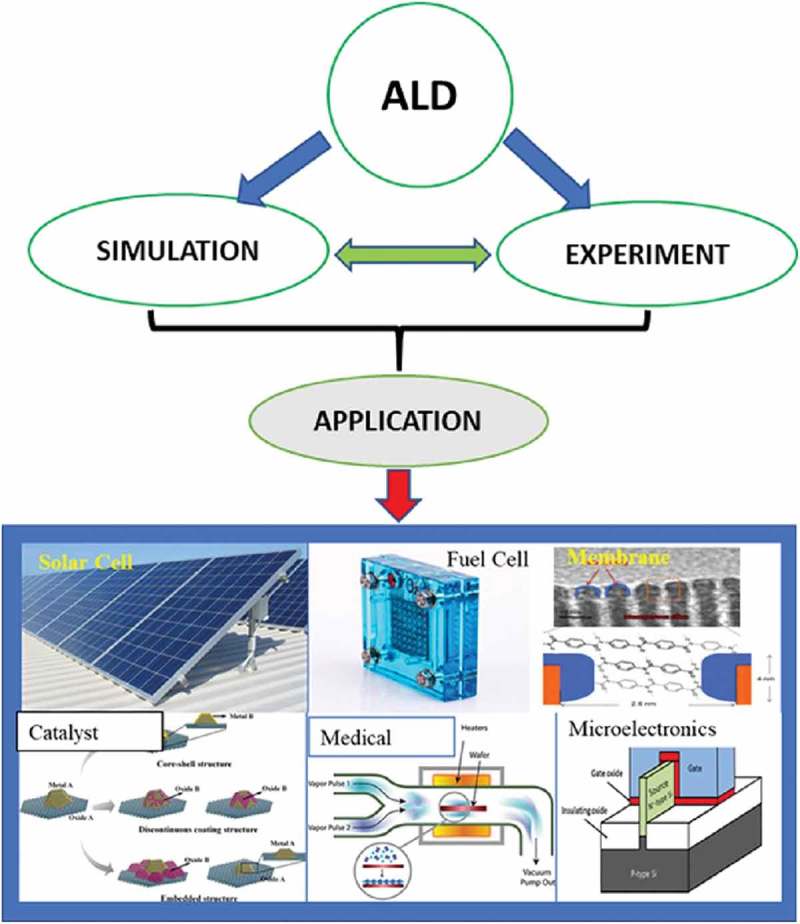

## 1. Introduction

### 1.1. Definition of ALD

Atomic layer deposition (ALD) is a thin film deposition technique where chemical precursors are sequentially introduced to the surface of a substrate where they chemically react directly with the surface to form sub-monolayers of film. As the name implies, it is fundamentally atomic in nature and results in the precise deposition of films on the surface of a selected substrate at the atomic scale.

### 1.2. Background

The concept of ALD was first introduced by Prof. V.B. Aleskovskii in his PhD work in 1952 [1]. The work was consolidated in another work on the process together with Prof. Kolt’sov in 1960, with which they published the principles of ALD with the title of ‘Molecular Layering’ [1].

In 1970, Dr. Tuomo Suntola along with other researchers developed an industrial thin film deposition technology and called it ‘atomic layer epitaxy’ (ALE) [2]. This technology found its first application in thin-film electroluminescent (TFEL) flat panel display [2], and the technology and materials selection has grown over years and find applications in photovoltaics, catalysis, semiconductor devices and many others [3].

ALD occurs by chemical reactions of two or more precursors injected alternately into a chamber where a substrate is placed at a given temperature and pressure to enable materials deposition on the substrate’s surface layer by layer [4]. Unlike chemical vapour deposition (CVD) and other similar deposition methods, in ALD the precursors are not pumped simultaneously, they are pulsed sequentially. Although there are similarities between ALD and CVD, the distinction lies in the self-limiting characteristics for precursor adsorption, alternate and sequential introduction of the precursors and reactants [5].

**Figure 1.**
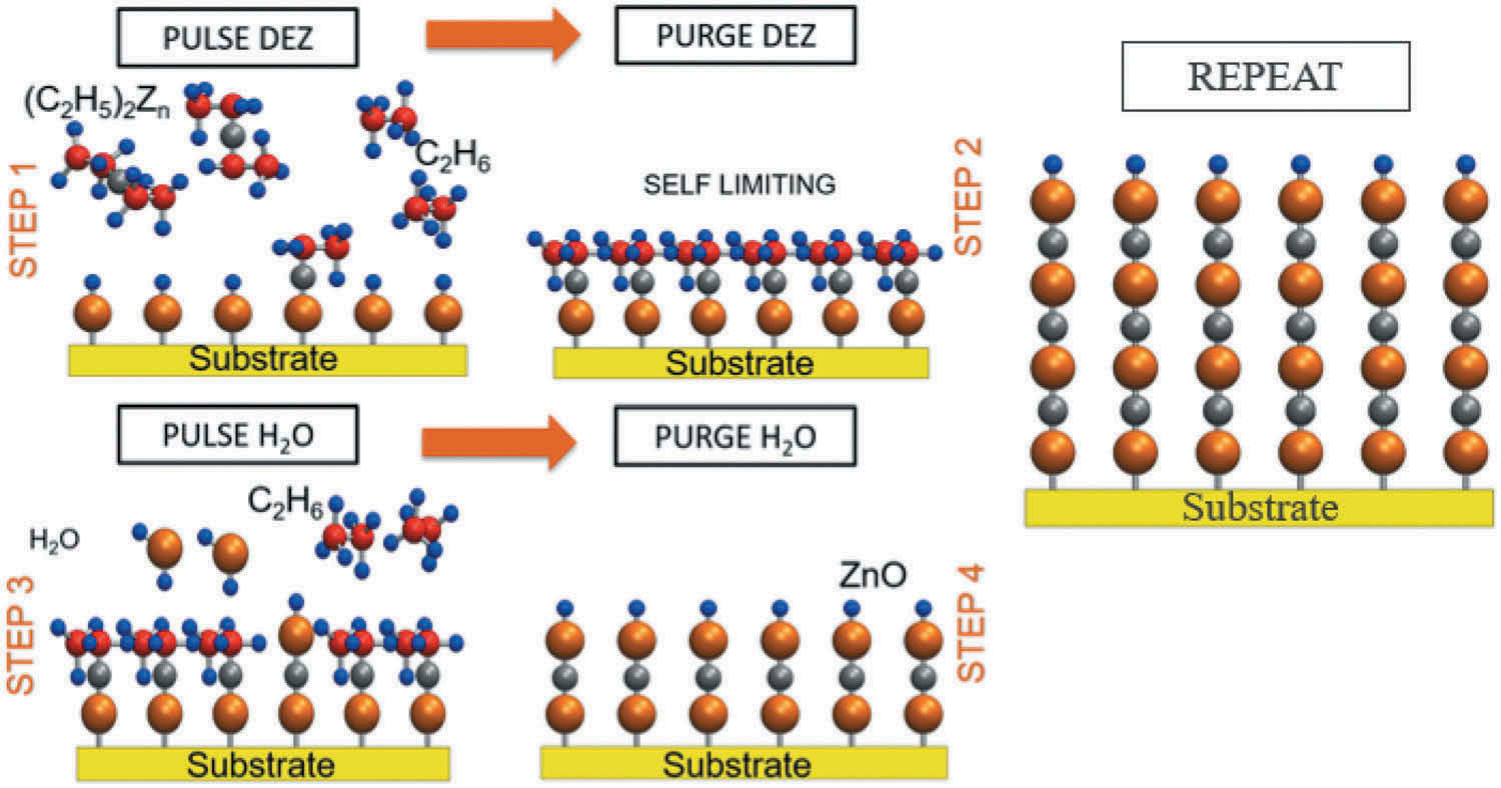
Illustration of ALD for ZnO thin film deposition. Adapted with permission from [11].

In addition, ALD as a thin film deposition method is becoming attractive because of its unique uniform deposition and conformal films on complex three-dimensional surfaces [6,7]. Thin film deposition can be divided into three main categories: the liquid, gas and solid based deposition methods which include but are not limited to physical vapour deposition (PVD) and the chemical vapour deposition (CVD). ALD can be counted as the most advanced version of the traditional CVD process [8]. In the ALD process, alternative pulses of precursor vapour and purge gas are introduced into the reactor, resulting in thin film growth due to self-saturating reactions with accessible surface groups, leading to a self-limited growth of a (sub) monolayer of material [9]. The process of ZnO thin film deposition by an ALD process is illustrated in Figure 1. As it is seen in Figure 1, the ALD process for two precursors are usually used in depositing metal oxide films; one is the metal source and the other is the oxygen source (oxidant) [10].

The synthesis of aluminium oxide (Al_2_O_3_) thin film is another example of an ALD process where trimethyl aluminium (TMA) and water in the thermal ALD reactor have been used to create a thin film [11–13]. The steps of a single cycle deposition process are as follows:

1. Exposure of the first precursor in the reactor chamber to form a layer on the substrate
2. Purge the excess first precursor and the by-products
3. Exposure of the second precursor
4. Purge or evacuation of the excess second precursor and by-products
5. The process is repeated until the required film thickness is achieved.

**Figure 2.**
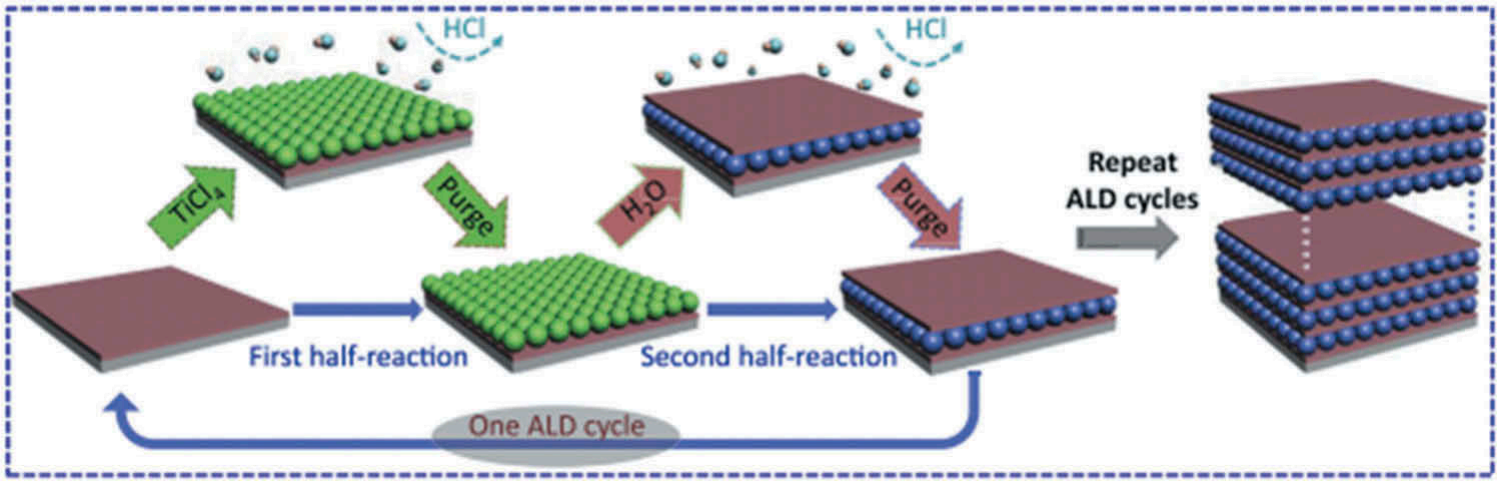
A model ALD process for depositing TiO_2_ on hydroxyl groups functionalized substrate using TiCl_4_ and H_2_O as precursors. Adapted with permission from [13], copyright Elsevier 2017.

The above processes are demonstrated in Figure 2.

This review looks at the progress in ALD in light of the process and product development in both areas of computational and experimental methodologies in this field to date. This will also provide insights into the outlook of this emerging technology.

### 1.3. Thin film applications

Thin film can be defined as a layer of material that its thickness ranges from nanometre to several micrometres. Thin film can be produced using varieties of techniques which are based on either physical or chemical processes. The thin film coating finds applications in many fields such as semiconductors [5,14–18], which enables the design of smaller semiconductor for a smaller device while performing better with higher energy efficiency. Application of thin film in fabricating batteries has also been reported to improve the efficiency of the batteries [13,19–28]. The solar industry is another industry which vastly relies on thin film technology as thin film is a potential alternative to present solar panels since it poses advantages such as easy production and higher flexibility [20,27,29–35]. Thin film finds another application in water purification and it can reduce the size of purification unit and also increases the efficiency such as in membrane technology and also fabrication of photocatalyst thin film for water treatment [36–43]. Thin film is very useful in wastewater treatment where the application of nanomaterial as a photocatalyst for pollution abatement is needed [44–47]. Applications of a thin film instead of nanopowder have been reported to be an effective method which immobilizes the nanomaterial on a surface of substrates and avoid the cumbersome separation of nanosize materials form water and wastewater. Air purification is another area where nanomaterial can be used as thin film [48,49]. Automobile industries can be accounted as another big applicant of thin film as it finds application in nearly every part of it, for example, applying thin film can reduce the size and improve the life of different parts [50]. Thin film has a huge application on medicine and medical devices, for instance, it can reduce the size of medical devices and make their application easier, more durable and secure [40,51–53].

### 1.4. Comparison of ALD with other coating techniques

There are several studies that have compared ALD with other film deposition techniques. Here we have summarized these comparisons in tabulated forms. Table 1 presents different types of film deposition methods, their advantages and applications of each technique.

**Table 1.** Different types of film deposition methods (adapted from [54]), the sputtering was adapted from [55] while the breath figure method was adapted from [56], with permission.

Method	Description and types	Advantages	Applications
*Electroplating*	Film formation from chemicals in electrolytic solution placed onto substrate surface with a seed layer on top	Corrosion resistance, decoration, mechanical characteristics improvement, protection barriers, electrical conduction and heat resistance	Metal plating, corrosion resistance, decoration, mechanical characteristics improvement, friction reduction, protection barriers, improved electrical conductivity, heat resistance and radiation protection etc.
*Spin coating*	Film formation from chemical reaction between liquid-phase sources (often sol–gel) applied onto surface of substrate while spinning	Simplicity and ease of set up, low cost and fast operating system	Photoresists, insulators, organic semiconductors, synthetic metals, nanomaterials, metal and metal oxide precursors, transparent conductive oxides, optical mirrors, magnetic disk for data storage, solar cells etc.
*Sputtering*	A process of deposition of materials because of bombardment of targets by high energy particles ejected from a source	Deposit a wide variety of metal and metal oxide nanoparticles (NPs) and nanoclusters (NCs), insulators, alloys and composites, and even organic compounds	Silicon wafer, solar panel or optical device, catalysis
*Breath figure*	A self-assembly process that results in a honeycomb-structured films with micro-pores arranged in honeycomb shape usually formed by water microdroplets condensed on a cool surface from warm, humid air like breath	It is simple and applicable to a wide variety of materials with highly organized honeycomb-like porous surface	Optics, photonics, surface science, biotechnology, and regenerative medicine
*Thermal oxidation*	Film formation by thermal oxidation of the substrate	Slow oxidation rate, good control of the oxide thickness and high values of breakdown field	Semiconductor industry, transistors, photoresistors, capacitors and field oxides, etc.
*Physical vapour deposition* (*PVD*)	Film formation by condensation of gasified source material, directly transported from source to substrate through the gas phase: Evaporation (thermal, E-beam), Molecular beam epitaxy (MBE), Pulsed laser deposition (PLD), Reactive PVD, Sputtering (DC, DC magnetron, RF)	Atomic level control of chemical composition, film thickness, and transition sharpness	Fuel cells, batteries, microelectronics, optical and conducting surfaces, etc.
*Chemical vapour deposition* (*CVD*)	Film formation by chemical reaction between mixed gaseous source materials on a substrate surface using: Atmospheric-pressure CVD (APCVD), Low-pressure CVD (LPCVD), Plasma-enhanced CVD (PECVD), Metal-organic CVD (MOCVD)	High growth rates, good reproducibility, epitaxial films growth, good film quality, conformal step coverage	Microelectronics, solar cells, fuel cells, batteries, etc.
*Atomic layer deposition* (*ALD*)	A sub-class of CVD with film formation via sequential cycling of self-limiting chemical half-reactions on the substrate surface. Each reaction cycle accounts for the deposition of a (sub) monolayer. The reaction can be activated by thermal energy or plasma enhancement. They can be categorized as: Thermal ALD, Plasma-enhanced ALD (PEALD), Spatial ALD (S-ALD)	High quality films, conformality, uniformity, step coverage	Fuel cells, desalination, microelectronics, capacitors, oxides, catalysts, etc.

In the industry, the PVD and CVD have been the popular deposition methods, however, ALD has been recognized as the leading emerging technology as nanometre-size layer thickness or pinhole free layers are becoming more important [54].

In comparison to CVD which relies on high temperatures to decompose the precursor at the substrate surface, ALD processes are performed at lower temperatures. The growth rate of ALD is related to the precursor’s flux at the substrate. It is complicated to determine the growth rate and it often operates within the temperature window, the point where the temperature and concentration of the precursor per pulse, and the purge times are balanced and the growth per cycle (GPC) is stable [54].

Table 2 provides the general overview and physical properties of the thin film deposited by the ALD process in comparison to other thin-film fabrication techniques which are similar to ALD. In this study, the authors have compared different deposition techniques based on the design parameters. As presented in Table 2, ALD has noteworthy advantages such as uniformity and control of deposition. However, due to its inherent layer by layer deposition, the deposition rate in the ALD process is low, which is the major drawback of this process.

**Table 2.** Comparison of thin film deposition techniques which are similar to ALD (Adapted with permission from Elsevier 2017 [57]).

Property	Deposition Technique					
	CVD	MBE	ALD	PLD	Evaporate	Sputtering
Deposition Rate	Good	Fair	Poor	Good	Good	Good
Film density	Good	Good	Good	Good	Fair	Good
Lack of pinholes	Good	Good	Good	Fair	Fair	Fair
Thickness uniformity	Good	Fair	Good	Fair	Fair	Good
Sharp dopant profiles	Fair	Good	Good	Varies	Good	Poor
Step coverage	Varies	Poor	Good	Poor	Poor	Poor
Sharp interfaces	Fair	Good	Good	Varies	Good	Poor
Low substrate temp.	Varies	Good	Good	Good	Good	Good
Smooth interfaces	Varies	Good	Good	Varies	Good	Varies
No plasma damage	Varies	Good	Good	Fair	Good	Poor

Abbreviations: Chemical vapour deposition (CVD), Molecular beam epitaxy (MBE), Atomic layer deposition (ALD), Pulsed layer deposition (PLD)

### 1.5. Advantages and disadvantages of ALD

The outstanding feature of ALD in comparison to other deposition techniques such as CVD and PVD is the self-limiting chemisorption of precursors in each half-cycle. This makes ALD unique in sub-nanometre film thickness and conformality control, offering next to (nearly) equal growth-per-cycle values for identical precursors in different equipment [54].

Table 3 presents the main advantages and disadvantages of the ALD process. ALD can create high-quality film with conformality and most importantly it works at low temperatures. ALD is exceptionally effective at coating surfaces that exhibit ultra-high aspect ratio topographies, as well as surfaces requiring multilayer films with good quality interfaces [54]. However, ALD is facing some challenges as mentioned in Table 3. The ALD process is a high precision process and this often leads to high precursor gas usage and energy. Approximately 60% precursor dosage is wasted in the ALD process which implies that most of the energy has been wasted as well as the concomitant labour. This low material utilization efficiency is shown to be about 50.4% of TMA deposited on wafers as revealed by experiments [55,56]. For instance, in the Al_2_O_3_ ALD process, ~4.09 MJ energy is consumed for the deposition of a film with 30 nm thickness and this shows the energy-intensive nature of the ALD process and invariable energy wastage [56,57]. In a previous study on nanoparticle emissions, Al_2_O_3_ ALD process shows that the total nanoparticle emissions with the diameter of less than 100 nm are in the range of 6.0 × 10^5^ and 2.5 × 10^6^ particles in 25 cycles of Al_2_O_3_ ALD process [58]. Another drawback in ALD for commercial use is the cost-effectiveness which is principally due to its deposition rate; however, this challenge has been partially overcome using spatial atmospheric ALD [58].

**Table 3.** Advantages and disadvantages of ALD.

Advantages	Disadvantages
High-quality films Control of the film thickness Excellent repeatability High film density Amorphous or crystalline film Ultra-thin films Conformality Excellent 3D conformality Large area thickness uniformity Atomically flat and smooth surface coating Challenging Substrates Gentle deposition process for sensitive substrates Low temperature and stress Excellent adhesion Coats Teflon Low-temperature processing Stoichiometric control Inherent film quality associated with self-limiting Self-assembled nature of the ALD mechanism Multilayer	The time required for the chemical reactions The economic viability Very high material waste rate Very high energy waste rate Intensive nature of the ALD process Nano-particle emissions

### 1.6. Complex 3D nanostructures

Owing to its conformal and self-limiting surface deposition, ALD has shown premier advantages to fabricate 3D complex nanostructures. A variety of materials such as semiconductors, magnetic materials, noble metals, and insulators are being fabricated using ALD to form 3D complex nanostructures, which are promising in the applications of high-performance transistors, nano-structures, energy storages and conversion.

Usually, ALD is used to fabricate complex 3D nano-structures [59]. For instance, surface modification of nanoporous materials is being realized through ALD coating with excellent step coverage inside of the porous structures. Such kind of structures with large surface areas with ALD-coated wide-bandgap semiconducting oxides, transparent conducting oxides, and ion conducting oxides have been used for solar cells, lithium-ion batteries, and solid oxide fuel cells [59]. The following table (Table 4) summarized the materials, reactants and templates used in ALD coating of nano-porous structures.

**Table 4.** Materials, reactants and templates used in ALD coating of nano-porous structures. Republished with permission from [59].

Materials	Reactants	Temperature	Template
TiO_2_	Ti[OCH(CH_3_)_2_]_4_/H_2_O	140 °C	Polycarbonate
ZrO_2_	Zr[OCH(CH_3_)_3_]_4_		
TiO_2_	TiCl_4_/H_2_O	105 ^o^C	AAO
ZnO	Zn(C_2_H_5_)_2_/H_2_O	200 ^o^C	AAO
ITO	InCp/O_3_ Tetrakis(dimethylamino)tin/H_2_O_2_	275 ^o^C	AAO
Ru	Ru(EtCp)_2_/O_2_	300 ^o^C	AAO
SiO_2_	H_2_N(CH_2_)_3_Si(OCH_2_CH_3_)_3_/H_2_O/O_3_	150 ^o^C	AAO
Fe_2_O_3_	Fe_2_(OBu)_6_/H_2_O	130 ^o^C − 170 ^o^C	AAO
Fe_2_O_3_	Fe(Cp)_2_/O_3_	230 ^o^C	AAO
Fe_2_O_3_ + Fe_3_O_4_	Fe(Cp)_2_/O_2_	350 ^o^C − 500 ^o^C	AAO
ZnS	Zn(C_2_H_5_)_2_/H_2_S	120 ^o^C	AAO
Sb_2_O_5_	(Sb(NMe_2_)_3_)/O_3_	120 ^o^C	AAO
Sb_2_S_3_	(Sb(NMe_2_)_3_)/H_2_S	120 ^o^C	AAO
Nb_2_O_3_	NbI_5_/O_3_	320 ^o^C	AAO

ITO stands for Indium tin oxide, AAO stands for anodic aluminium oxide, Ru(EtCp)_2_ stands for bis(ethycyclopentadienyl)ruthenium, Sb(NMe_2_)_3_ stands for Tris(dimethylamido)antimony(III)

Using diatoms as templates, Losic et al. [60] demonstrated coating of ultrathin films of titanium oxide (TiO_2_) by using titanium chloride (TiCl_4_) and water to modify the pore size. Such structures show huge potential in membrane applications in microfluidic systems.

**Figure 3.**
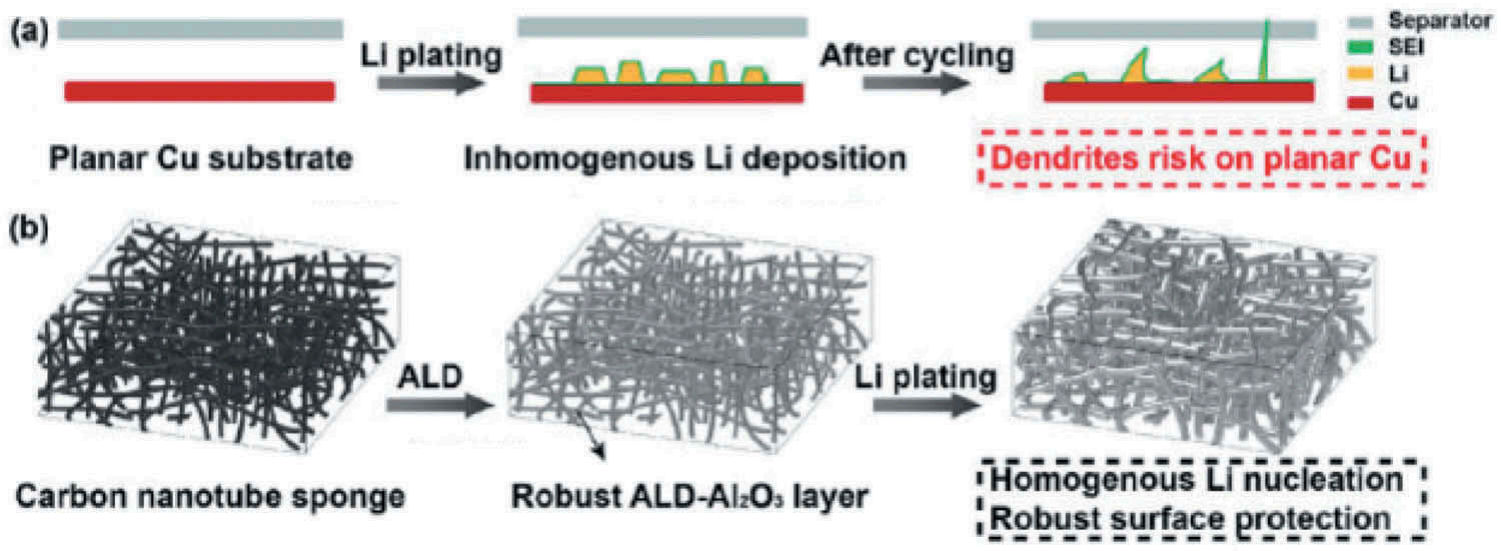
Schematic illustration of the Li deposition process on planar Cu and 3D ALD-CNTS substrates. (a) Inhomogeneous Li deposition resulted in the formation of Li dendrites, which punctured the separator after repeated cycles. (b) A high-specific-surface-area CNTS network with a robust Al_2_O_3_ layer on the surface ensures homogenous Li nucleation during the Li plating process and forms a stable, dendrite-free Li metal anode.

In their work, Zhang et al. [61] used ALD to coat a layer of Al_2_O_3_ on top of Li depositions on a 3D carbon nanotube sponge (CNTS) structure to protects the Li metal electrode/electrolyte interface in Li-ion-based batteries as illustrated in Figure 3. The ALD-deposited Al_2_O_3_ layer provided desirable chemical stability and high mechanical strength for Li@ALD-CNTS electrode.

**Figure 4.**
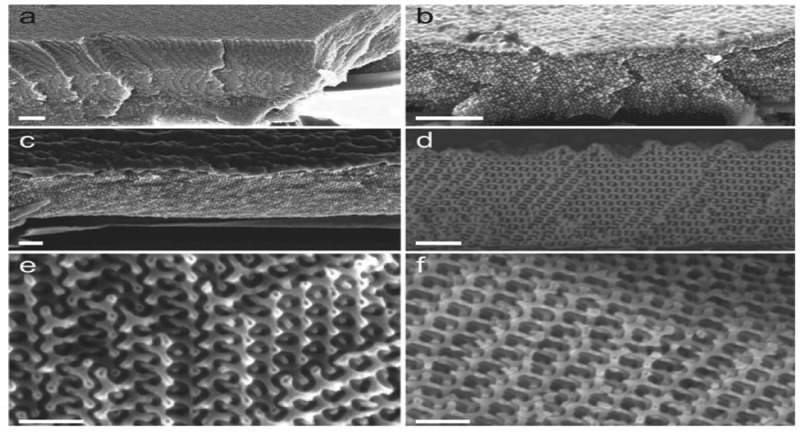
Side-view scanning electron microscopy (SEM) images at a 45° angle of gyroid replication into ZnO: (a) gyroid polystyrene template, (b) as-deposited ZnO-PS hybrid, and (c) ZnO gyroid after annealing at 550 °C. (d,e,f) Different faces of the ZnO gyroid shown in (c). The scale bars correspond to 1 μm for (a) and (b), 400 nm for (c) and (d), 200 nm for (e) and (f).

Kim et al. [62] reported a method of using ALD to synthesize crystalline 3D mesoporous ZnO networks based on a self-assembled block copolymer template. Two 3D mesoporous morphologies including a periodic gyroid structure and a random worm-like morphology were fabricated as shown in Figure 4. Metrological examinations showed that the ALD deposited films were uniform in terms of pore size and material composition. Such complex 3D structures are attractive for hybrid photovoltaic applications such as fabricating ZnO solar cells.

More applications of using ALD for self-limiting surface modifications to fabricate 3D nanostructures used in energy storage and conversion were reviewed and summarized in the review [63]. For instance, using ALD, copper indium sulfide (CuInS_2_) was deposited into a porous TiO_2_ electrode to improve the responsiveness of solar cell compared to the conventional dye-sensitized cells [63].

However, due to the complex structures of the templates, such as porous structures, reactant molecules tend to be confined inside of such complex structures. The precursor flow will be hindered, and hence the reactions can be different from those reactions on a planar surface, in terms of both mechanism of nucleation and growth of the film [63]. To achieve desirable conformal surface coatings, the optimization of ALD process on the 3D structures will require a more detailed understanding of their physical, chemical, and electrochemical properties in order to precisely tune the process parameters.

The specific challenges for the characterization of 3D nanoarchitectures were summarized in the reference [63]. In-lab materials science analyses provide a reasonable understanding of the character of a given nanostructures to optimize synthetic and fabrication strategies. Such metrological techniques include simultaneous differential scanning calorimetry and thermogravimetric analysis to guide thermal treatment of the structure, X-Ray diffraction analysis to provide information on the ‘bulk’ structure of the solid, and physisorptive analyses to provide surface area, pore volume and the distribution of pore sizes [63].

Samples could be further characterized by field emission scanning electron microscopy, transmission electron microscopy, small-angle neutron scattering, X-ray photoelectron spectroscopy and vibrational spectroscopy, Fourier-transform infrared spectroscopy, and solid-state nuclear magnetic resonance [63].

## 2. ALD processes

There are different ALD process modes but the most widely used ALD modes are thermal ALD and plasma-enhanced ALD (PEALD). In addition, plasma-assisted or radical-enhanced ALD are also referred to as PEALD. Thermal ALD is mainly a surface-driven process, which occurs exclusively through surface reactions, thus enabling good thickness control and conformality irrespective of the substrate geometry and reactor design. Thermal ALD processes require relatively high temperatures (typically 150–350 °C); this causes a limitation on their applications which can be addressed by PEALD [64].

PEALD, also referred to as plasma-assisted ALD (PA-ALD), has high reactivity of the plasma species which enables the reduction of the deposition temperatures without compromising the film quality. It is appreciated especially when the thin films are deposited on temperature-sensitive materials. PEALD can also lead to improved material properties. Furthermore, the reactivity of the plasma facilitates the use of a wider selection of precursors and enables the deposition of materials that are challenging or inaccessible by the means of thermal ALD [65].

Increasing the surface area and volume of an ALD reactor leads to longer pulse and purge time resulting in lower throughput. This is improved by using spatial ALD (SALD), which eliminates the pulse purge chambers with the spatially resolved head by exposing the substrate to a specific precursor based on location, though the quality of the thin film would have been decreased [58]. SALD technique can achieve deposition rates of around 3600 nm/h [7]. Table 4 gives the advantages and disadvantages of plasma enhanced atomic layer deposition. PEALD allows deposition at relatively low temperatures and film properties are better than the ones in thermal ALD [66]. PEALD also provides highly reactive species or catalysts that enhance the reaction thus enabling the use of lower temperatures, as well as a broader range of substrates and precursors for deposition [67]. Therefore, PEALD is useful for the growth of film on the substrates that are heat sensitive. PEALD can also produce highly pure films than the films produced by thermal ALD [68]. Other advantages of the PEALD are the wider choice of precursors, higher growth rate, and process versatility [69]. Table 5 has summarized the advantages and disadvantages of the PEALD method.

**Table 5.** Advantages and disadvantages of PEALD.

Advantages	Disadvantages
Low deposition temperature	Limited conformality
Higher reactivity (shorter deposition times)	More complicated reactor designs
Higher film purity	More complicated reaction chemistry
Wide range of chemistry possible	
Denser films	Potentially poor conformality
Higher throughput	Lower throughput
*In situ *plasma treatment	Damage to films
Lower impurity	Additional growth parameter

Despite the advantages of the process, PEALD also precipitates undesired side-reactions. To derive the maximum advantages, the ALD process should be within this optimum temperature window, else poor growth rates, slow reaction kinetics or precursor condensation and thermal decomposition could occur [7]. As mentioned earlier, the ‘ALD temperature window’ is the temperature range where the growth is saturated, and it depends on the specific ALD process.

**Figure 5.**
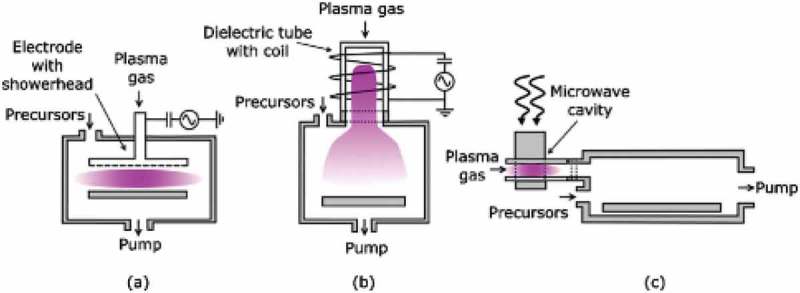
Schematic representation of the three different types of plasma-assisted atomic layer deposition that can be distinguished: (a) direct plasma (b) remote plasma, and (c) radical enhanced. For each type different hardware configurations and plasma sources.

Although PEALD has limited conformity and reactor designs are more complicated, its low deposition temperature makes it valuable for some applications such as capacitors, DRAM, etc. Figure 5 illustrates three types of plasma sources for PEALD at low pressures. The first configuration (a) is called the ’direct plasma’, because the substrate is exposed to high energy ions or the deposition surface is in contact with the active plasma. The second configuration (b) is known as a remote plasma system-the contact between plasma and substrate as well as the mild ion energies distinguish remote plasma ALD from radical-enhanced and then the ’radical enhanced’ or ’radical assisted’ (Figure 5(c)) ALD. Radical enhanced ALD has an advantage that the substrate or growing film are not damaged by energetic plasma species [68] and only the neutral plasma species participate in the deposition process.

Recently a branch of ALD, called the photo-assisted ALD or UV-enhanced ALD, has been developed. There the illumination by UV light is adapted as a part of the ALD cycle. UV exposure has been shown to enhance the surface reactions and lead to improved film properties [65].

## 3. Methods for studying ALD

Research study on the ALD process is growing into two main categories: numerical and experimental. In the experimental studies, researchers have improved the deposition of the films by adjusting the conditions such as; pressure, temperature, purge time, concentration, etc [4]. The ALD process has quite extensive materials range to choose from however not all materials can be used; hence, the need for simulation to determine and predict which effective reaction pathways are necessary.

Due to its atomic scale deposition which is relative to the feature and reactor scale, the ALD numerical method inherently involves multi-scale analysis. The multi-scale process includes atomic bond formation, species chemisorption/adsorption, chemical kinetics and film deposition. It also includes a reactor-scale that involves material selections/interactions, geometry effects, and fluid and energy transport on a macroscopic level [70–77]. A challenge of the feature and reactor scales is that neither addresses the other limitations placed on the prediction capability of the deposition process over the substrates [78–81].

The mesoscopic scale is introduced as the third scale to assist in this problem. At the mesoscopic scale simulation, the continuum laws are still valid. This scale also obtains its net fluxes from the feature densities. The mesoscopic scale couples-decouples between the scale processes by evaluating an element on the reactor scale grid [78–81]. A more detailed description of the mesoscopic scale will be illustrated in the next section.

Good exposure of the substrate surface is effective to enhance saturation in the ALD process. High sticking probability can be achieved by modification of the surface or by controlling the chemistry of the reaction. To achieve true ALD growth, the purging time between the precursors’ exposure time should be sufficient; if not there might be a CVD mode growth due to gas phase precursors mixing, which will lead to poor conformality due to the gas phase reaction [5]. Time for getting saturation is much longer for heavier molecules. The molecular flux is inversely proportional to the square root of the molecular weight.

Remmers et al. [82] were able to develop a procedure using the Gauss–Jordan factorization to separate the modes of thin film deposition with particular interest on ALD because of the cyclic dynamic behaviour and complexity in defining the reactions process on amorphous films. They represented the growth process in the modelling by formulating the full set of adsorption, desorption, and surface reactions into two reaction mechanism cycles, each of which can produce an independent limit-cycle solution. According to Travis and Adomaitis [52], the deposition rate in ALD largely depends on the instantaneous growth state of the surface and this changes throughout each exposure and purge time.

In comparing modelled results and experimental results of the ALD process, Jones et al. [83] set up a model to predict the chemical species in a reactor as a function of time and space to give an optimal ALD process both the experimental and modelled results had a good agreement. The modelled result confirmed that the increased dissociation rate decreases the overall concentration in the chamber irrespective of the pressure. Scale-up analysis of continuous cross-flow atomic layer deposition reactor designs was reported by Holmqvist et al. [84]. They identified the optimal scaling rules in maximizing the utilization of precursors in the system for a fixed growth rate and relative uniformity.

### 3.1 Numerical methods

#### 3.1.1. Knudsen number

In ALD simulation by Knudsen number method, both very large and very small Knudsen numbers ($K_{n}$) can coexist [76]. The Knudsen number depends on the gas flows pressure, temperature, and the characteristic reactor dimensions. It defines the ratio of the mean free path and the characteristic length. Likewise, it can also be defined as the molecule-wall to molecule-molecule collisions. The Knudsen number is used to indicate which formulation should be used for a specific situation as the mean free path becomes comparable with, or even larger than, the characteristic length (high $K_{n}$ number), at which the particle nature of matter must be clearly accounted for, since the continuum assumption breaks down, particularly in the area of micro-scale or nano-scale processes [85]. A flow type can be characterized as a continuum with $K_{n}$ < 0.1, free molecular where $K_{n}$ ≥ 10, or transitional in which 10 > $K_{n}$ > 0.1 [86,87]. To elaborate, the Navier-Stokes equations with no-slip boundary conditions can be used to simulate very small Knudsen numbers (continuum flows) or utilize statistical mechanics at large Knudsen numbers where the Navier-Stokes equation no longer upholds (molecular flows) [88,89]. At intermediate values of *K_n_*, kinetic models based on the Boltzmann transport equation capture the influence of both transport and collisions among the molecules; this is called the transition regime.

The Knudsen number can be expressed as:

$$K_{n}=\frac{\lambda }{d}$$

The mean free path (λ) can be found as

$$\lambda =\frac{k_{B}T}{p\sqrt{2}\pi d_{g}^{2}}$$

where p is the fluid pressure, $d_{g}$ the effective diameter of the molecule, $k_{B}$ the Boltzmann constant ($1.3807x10^{-23}\;J/K)$, T the temperature of the fluid (K), and d the characteristic length [90].

**Figure 6.**
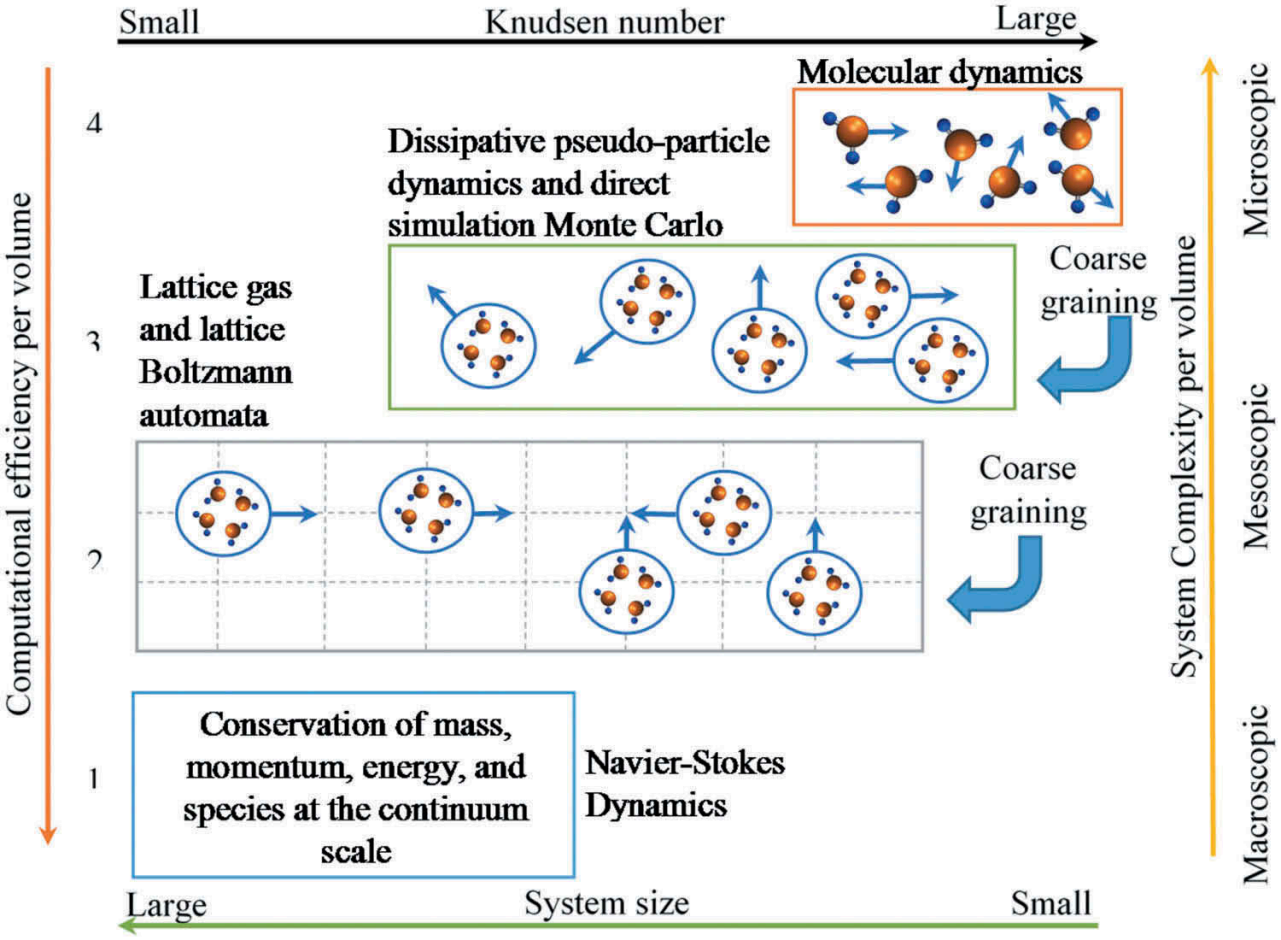
Various methods in describing fluid flow at different levels (modified with permission from Liao and Jen [91] and Coetzee and Jen [11]).

Along with the Knudsen number, other influential properties arise to limit and define the numerous simulation techniques that are used to quantify the ALD process. These properties include but not limited to computational efficiency, system complexity, and simulation size. In Figure 6, the relation between these properties and simulation methods are illustrated. It can be deduced from this illustration that as the assumption of a volume with no particle space in between moves (continuum approach) to a cluster of small particles (molecular dynamics (MD)). The Knudsen number and system complexity per volume will increase, although the computational efficiency per volume and system size will decrease. These models can range from very fundamental solutions of sets of simple interactions of particles (such as molecular dynamics) to systems in which the individual particles are replaced by continuum fluid elements estimations (such as the Navier-Stokes equations) [85]. This can be organised from the most fundamental level molecular dynamics (MD), Monte Carlo method, the Lattice Boltzmann Method, to the continuum approach.

#### 3.1.2. Monte Carlo

3D (three-dimensional) Monte Carlo model was used by Cremers et al. [92] to compare the deposition of an infinite array of holes and infinite arrays of pillars using atomic layer deposition on large 3D surface area. They observed that the required exposure to conformally coat an array of holes is determined by the height to width ratio of the individual holes and is independent of their spacing in the array. In studying the conformality of plasma enhanced ALD (PEALD) using the Monte Carlo method, Knoops et al. [93] used the recombination probability, the reaction probability and the diffusion rate of particles to observe the conformal deposition in high aspect ratio structures (step coverage). They identified three deposition positions: a reaction limited regime, a diffusion limited regime and a recombination limited regime. From their findings, conformal deposition can be achieved in high aspect ratio structures with a low value of recombination probability. Shirazi and Elliott [88] studied the atomistic kinetics of atomic layer deposition using the Monte Carlo method derived from density functional theory (DFT) [94]. They looked at the early stage adsorption of the ALD precursor, the surface proton kinetics, the steric effects, influence of remaining fragments on adsorption sites, densification of the precursor, migration, and cooperation of the precursors. They concluded that the essential chemistry of the ALD reactions depends on the local environment at the surface.

#### 3.1.3. Molecular dynamics

Molecular dynamics (MD) is a numerical method that uses Newton’s equations of motion for computationally simulating the movements of atoms and molecules. The techniques depend on the description of the interaction of molecules-force field and are extensively used in chemistry, physics, biophysics and biochemistry. MD more often is about developing quantitative predictions of molecular shape, sizes, flexibilities, the interactions with other molecules, its behaviour under pressure, and the relative frequency of one state or conformation compared to the others [95]. Using Newton’s equation of motions, the simulation calculates the forces between the atoms at each time step and updates the positions of the atoms at the following time step [96]. High costs and difficult chemical management associated with ALD studies have made more researchers to conduct numerical modelling of the ALD process to understand and study the operation process. In modelling, further insight into the ALD process is gained, hence minimizing the precursors’ inputs and wastes and also reduces the potential environmental impacts in future industrial productions [97].

As compared to other modelling methods, there are few studies using molecular dynamics for ALD modelling. In MD simulations the non-bonded interactions between atoms that are being considered and calculated using the Lennard-Jones potential parameters and the columbic potentials [96,98–100].

$$E=E_{vdW}+E_{Columbic}$$

Where

$$E=4\varepsilon \left[{\left(\frac{\sigma }{r}\right)}^{12}-{\left(\frac{\sigma }{r}\right)}^{6}\right]+\frac{q_{i}q_{j}}{\varepsilon_{d}r}$$

ε and σ are the well depth of potentials between any two atoms and the distance between any two atoms, respectively. The charges between the two atoms i and j are $q_{i}q_{j}$, r is the distance between atoms i and j, and $\varepsilon_{d}$ is the dielectric constant.

Computational software like reactive force-field (ReaxFF) [101] and DFT [102–104] can be used to gain further insight into the dynamics of the atomic layer deposition process [101]. ReaxFF interatomic potential is a powerful computational tool for exploring, developing and optimizing material properties [101]. ReaxFF is a force-field technique that uses the bond order concept in modelling the interactions in a chemical system [105]. While DFT is a computational method that derives properties of the molecule based on a determination of the electron density of the molecule. It calculates the molecular energy from the electron density, deliver a force field of high accuracy and relatively computational simplicity and efficiency.

Other software, such as LAMMPS [38,106–109], NAMD [53], GROMACS, CHARMM [53] and AMBER [95,100] is also used for molecular dynamics simulation. For instance, by using LAMMPS for molecular dynamics simulation, Hieranian et al. [109] demonstrated that a single layer of molybdenum disulfide (MoS_2_) can effectively separate ions from water. They investigated the desalination of water through (MoS_2_) as a function of chemistry, pore size, hydrostatic pressure and geometry. To have efficient desalination, the sizes of pores should be such that both the water filtration and ion rejection are optimized. In one hand large pores do not effectively reject ions while very small pores permeation rate is low. Atomic layer deposition is effective in addressing such situations. Hu et al. [110] used MD to study and predict the influence of the initial surface composition and process temperature on the roughness of the surface, the growth rate and growth mode of the film deposition. Timo and Kari [111] in the study of the atomic layer deposition of alumina by TMA–H_2_O-process, they used MD simulation to study the water reaction mechanism with alumina to obtain more insight on the surface mechanisms and energetics which according to them is essential in the design and optimization of the ALD process.

There are studies that provide more insight into molecular dynamics and have explained from basic to advance [99,112,113].

There are other softwares that have been developed with a graphical user interface (GUI) like QuantumATK [104,114] and Lammpsfe that can also be used for ALD molecular dynamics study.

#### 3.1.4. Computational fluid dynamics (CFD)

The computational fluid dynamics method (CFD) involves a continuum approach that has the smallest Knudsen number (Kn<0.1). Intrinsically, this ends with a numerical method that has the highest computational efficiency per volume. Due to this technique treating the fluid as a continuous medium, it illustrates a fully filled domain with no space between the molecule particles. The CFD method attempts to solve the ALD process numerically by applying the governing equations of conservation of mass, momentum, energy, and species transport [12,89,115–118]. These processes are presented by corresponding partial differential equations (PDEs) that are solved numerically on defined nodes in a mesh domain. However, in literature due to the complexity of this fabrication process, studies favoured and focused purely on the mechanical [87,119–122] or the chemistry aspects [52,123] separately. Although this is said, in the past few years researchers [4,13,77,89,116,117,124,125] have applied this technique to combine both mechanical and chemical aspects, in which the necessary chemical data is obtained through the Density Function Theory (DFT) chemical simulation techniques or by chemical reaction experimentation [126]. The DFT technique plays a crucial role in the CFD modelling method. The DFT technique incorporates the required data to simulate the heterogeneous and homogenous reactions within the ALD process, including distinguishing different chemical recipe adsorption/desorption kinetics properties [73,127]. The analysis by combined DFT/CFD is currently rare, however, gaining importance as ALD is moving forward into the industrial realm.

Finally, these properties are utilized within the governing equations by coupling and decoupling the chemical reactions source terms from the prior governing equations. These modelling steps governing equations are expressed as:

$$\frac{\partial \rho }{\partial t}+\nabla \rho \vec{v}=0\;\;$$

$$\frac{\partial \rho \vec{v}}{\partial t}+\nabla \left(\rho \vec{v}\vec{v}\right)=-\nabla P+\nabla \tau +\rho \vec{g}+\vec{F}$$

$$\frac{\partial \rho E}{\partial t}+\nabla \left(\vec{v}(\rho E+\rho \right))=\nabla \left(k_{eff}\nabla T-\sum^{h_{i}}J_{i}\right)+R_{r}$$

$$\frac{\partial \left(\rho Y_{i}\right)}{\partial t}+\nabla \rho \vec{v}Y_{i}=-\nabla \vec{J_{i}}+R_{i}$$

where *J, k_eff_* and *R* resemble the diffusion flux term, effective conductivity, and reaction source term, respectively.

The r^th^ irreversible surface reaction can be shown as the following general form:

$$\overset{N_{g}}{\underset{i=1}{\sum }}g_{i,r}^{\prime }G_{i}+\overset{N_{b}}{\underset{i=1}{\sum }}b_{i,r}^{\prime }B_{i}+\overset{N_{s}}{\underset{i=1}{\sum }}s_{i,r}^{\prime }S_{i}=\overset{N_{g}}{\underset{i=1}{\sum }}g_{i,r}^{{\;}^{\prime \prime }}G_{i}+\overset{N_{b}}{\underset{i=1}{\sum }}b_{i,r}^{{\;}^{\prime \prime }}B_{i}+\overset{N_{s}}{\underset{i=1}{\sum }}s_{i,r}^{{\;}^{\prime \prime }}S_{i}$$

where *G, B* and *S* correspond to the gaseous species, the bulk species, and the site species, respectively. The molar reaction rate for the irreversible surface reaction can be calculated as follows:

$$R_{r}=k_{f,r\;}(\overset{N_{g}}{\underset{i=1}{\prod }}{\left[C_{i}\right]}_{wall}^{\eta_{i,gr}^{\prime }})(\overset{N_{s}}{\underset{i=1}{\prod }}{\left[S_{j}\right]}_{wall}^{\eta_{i,sr}^{\prime }})-k_{b,r\;}(\overset{N_{g}}{\underset{i=1}{\prod }}{\left[C_{i}\right]}_{wall}^{\eta_{i,gr}^{{\;}^{\prime \prime }}})(\overset{N_{s}}{\underset{i=1}{\prod }}{\left[S_{j}\right]}_{wall}^{\eta_{i,sr}^{{\;}^{\prime \prime }}})$$

These equations can be utilized to derive properties of interest in the ALD process focusing on macroscopic parameters, such as the mass deposition rate (${\dot{m}}_{dep}$) that can be obtained at the substrate surface as:

$${\dot{m}}_{dep}=\overset{N_{b}}{\underset{i=1}{\sum }}M_{w,i}{\hat{R}}_{i,bulk}$$

Shaeri [89] utilised CFD technique to investigate the reactor inlet position, multi inlets and the effects of high substrate temperatures [10,89]. These studies were comparable to experimental results and illustrated the effects of the mass fraction, coverage, deposition rate and growth rate. Pan et al. [115] also focussed on examining the geometrical effect of gap size on a spatial ALD. They also investigated with experimental validation where the effect of concentration and pumping pressures, pulse/purge times, gap sizes, heating temperatures and flow rates were studied [4,115]. Deng et al. [128] attempted to optimise the ALD process by quantitatively analysing the temperature, precursor mass fractions, mass flow and pressure. They concluded that while higher temperature increased the growth rate and accelerated the surface deposition processes, it had little influence on precursor distribution in the reactor chamber. Intrinsically, their simulation and experimentation also revealed that gas velocity decided the mass flow rate and chamber pressure as the determinant factor for minimising the cycle time.

Holmqvist et al. [121] investigated the controlling parameters on the deposition of the ZnO thin film in a continuous cross-flow ALD reactor. The study oriented towards the optimisation and control of the film thickness profile by evaluating the impact of changing operating parameters such as the local coordinate variable, process cycles, and temperature. In a second study, Holmqvist et al. [120] showed that the model predictions of the spatially dependent film thickness profile were in good agreement with both calibration and validation experimental data, respectively, under a wide range of operating conditions.

To further understand the ALD process, the reactor geometrical design also plays a crucial role. These studies may reveal optimal parameter conditions, defect in the ALD recipe, chemical distribution and dosage requirements, thermal flow effect, among others. The studies by Shaeri [89] and Pan et al. [115] that were previously mentioned have been accomplished by means of utilising a typical Cambridge Nanotech Savanah S100 reactor. Most recently, some other reactor designs were analysed by utilizing CFD simulations. Work from Coetzee et al. [117] studied the internal behaviour of a Gemstar 6 square type ALD reactor. In their findings, they discussed the effect of flow in these reactors and the unique buffer layers formed on the substrate surface due to the injection sequence recipe for creating Al_2_O_3_ thin film. A study from Peltonen et al. [119] studied the Reynolds Number flow effect and injection arrangement that affects the precursor delivery in the Picuson R200 reactor. Their simulations point to the relatively strong variations in the flow fields between low and high Reynolds numbers. By investigation of the flow through their reactor geometry, the study revealed that while the increasing Reynolds number can reduce the required pulse time, no major gain is obtained in the total time sequence of the ALD-cycle. This is due to the purging remaining a slow process, especially in the case of the increased Reynolds number flow nearing turbulence. Additionally, the study confirmed that various reactor specific geometric designs and operating properties, influenced by the different flow patterns observed at different Reynolds numbers, play an important role in the gas flow distribution. However, their study ignored the chemical reaction behaviours at the substrate surface.

A study from Gakis et al. [129]. studied the unique flow properties of the Ultratech Fiji F200 reactor. Their model aimed into the investigation of the influence of their reactor geometry and process conditions on the gas flow, temperature fields, and on the species distribution on the heated substrate surface, by neglecting the chemical reactions process at the substrate. The study results illustrated the non-uniformity flow at the pulsing flow and the purging flow entering the reactor through its loading door that affected the temperature and reactants concentration on the substrate surface.

The substrate topology can be regarded as another critical research domain to understand the flow behaviour across the reactive substrate. However, the topology can cause resistance to uniform deposition film due to the nature of its complex shape. Considering that the future application of ALD would be found within these complex three-dimensional structures, the behaviour and time evolution within these complex topologies could be of great importance. Current studies from the authors [130,131] focus on the development of a fundamental understanding of the mechanistic behaviours within pores and trenches incorporated into the reactive substrate. The author's current studies report on the flow phenomena, mass fraction, surface coverage, deposition rate and conformal growth of Al_2_O_3_ thin film within trenches along the substrate. One of the studies is to develop model and analyses on the vertical shower head injected type two-dimensional reactors. The purpose of the study is to investigate the mechanistic change of the parameters if a system is operating with and without an additional exposure time. The flow behaviours within and along the trenches are clearly seen and reveal interesting dependent properties to the film growth phenomena. Moreover, the time evolution of the half-reaction surface adsorption process is observed and analysed, along with the major enhancement of the exposure time brings to the ALD process. A study from Olotu et al. [131] revealed the change of the ALD mechanistic behaviour over the previously mentioned substrate and reactor design with the change of pressure. Within his findings, the flow phenomena over the trenches with an introduced exposure time revealed influential mechanistic flow patterns along with different time evolution requirements to create conformal film growth at different pressures. These and future studies are currently being investigated by the authors that incorporate different ALD film recipes, adsorption/desorption kinetics, reactor and substrate mass/fluid flow behaviours, system optimization and the creation of improved ALD processes, among others.

It is worth noting that the analysis technique of the process is scarce in most of the other ALD reactor designs and manufacturers. Due to the different designs of these reactors, future designs in the optimization of the ALD process will need to be similarly investigated to obtain optimal thin film fabrication processes for the numerous types of thin films ALD recipes.

### 3.2. Experimental methods

ALD grown materials which include oxides, nitrides, sulfides, pure elements and inorganic compounds are realized through the pulse/purge process in ALD reactors. The ALD film deposition methods experimentally depend on the factors such as the nature of the substrate, the deposited materials and the reactor design. The growth rate in ALD is strongly dependent on the aspect ratio of the substrate and the reactor design. Increase in the surface area and volume of an ALD reactor leads to an increase in pulsing and purging time. Substrate structures with high aspect ratio require longer pulsing and purging time for the gases to disperse evenly into the trenches and the three-dimensional features. Spatial ALD was introduced to overcome the longer pulsing and purging time by replacing the pulse/purge chambers spatially revolving heads. This exposes the substrate to a specific gas precursor-based location within the reactor [7]. Numerous reactants/precursors are used in ALD. Non-metal and metal precursors react differently and show different features so are organic compounds [8].

#### 3.2.1. Chemical supply

Wide range of materials can be grown by ALD process. These materials find applications in areas which include semiconductors, metals, insulators, organic and inorganic compounds. Table 6 tabulates materials synthesized by ALD in recent studies based on temperature, film thickness, method and application.

**Table 6.** Materials grown by ALD process.

Material	Precursor	Purge	Temperature	Film thickness	Method	Application	Ref
TiO_2_	TTIP (Titanium (IV)	N_2_	90	103 nm/cycle	Low-temperature	Cotton fabrics	[132]
SrTiO_3_	Ti(CpMe_5_) (OMe)_3,_ O_3_	N_2_	370	0.05 nm/cycle		Capacitor	[133]
TiO_2_	TDMA, H_2_O isopropoxide, DI		150	0.055 nm/cycle	Thermal	Catalysts membranes, dye-sensitized solar cells, batteries sensor	[134]
TiO_2_	SiO_2,_ [(EtCp)Ti(NMe_2_)_3_ Et = CH_2_CH_3_]	O_3_, O_2_	250–300		Thermal	MicroelectronicsDevices	[135]
Ge–Sb–Se–Te (GSST)	Sb (OC_2_H_5_)_3_ and [(CH_3_)_3_Si]_2_	Ar	70		Low-temperature	PcRAM	[136]
SnO*_x_*	Tetrakis(dimethylamino)tin(IV) and H_2_O	N_2_	80	(0.15 ± 0.01) nm/cycle	Spatial Atmospheric Pressure	Solar cell	[137]
SnS_2_	Sn(OAc)_4_ and H_2_S	N_2_	150–250	0.17 Å/cycle	Low‐temperature	Electronicscatalysis	[138]
TiO_2_	Titaniumtetraisopropoxide (TTIP) and H_2_O	N_2_	200	0.02 nm/cycle	Thermal	Nano wires	[139]
Si-O-Si(CH_3_)_3_	HMDS ((CH_3_)_3_-Si-N-Si(CH_3_)_3_) + TMCS (Cl-Si(CH_3_)_3_) and H_2_O	N_2_	180		Plasma	Carbon separation, capture	[140]
Sb_2_Se_3,_ TiO_2_ and Pt	Titanium(IV) tetraisopropoxide (TTIP), and H_2_O	N_2_	160			Solar energy conversion	[141]
Al_2_O_3_ and TiO_2_	TiCl_4,_ TMA OH, O_2_		150 and 250		Low-Temperature	Filtration, gas storage, and catalysis	[142]
Al_2_O_3_			150, 200, and 250			Nano-electronic devices	[143]
Al_2_O_3_	[Al(NMe_2_)_2_(DMP)], [Al(NEt_2_)_2_(DMP) [Al(N*i*Pr_2_)_2_(DMP), H20		100 and 180		Thermal and plasma ALD	Microelectronics, organic electronics, solar cells	[168]
V_2_O_5_	vanadium triisopropoxide (VTIP)	N_2_	150 to 300		Thermal	Capacitors	[202]
Ru	Ru(DMBD)(CO)_3_ and O_2_	N_2_	290 to 320	0.067 nm/cycle	Thermal	Transistors, capacitors	[144]
RuO_2_	Ru(DMBD)(CO)_3_ and O_2_	N_2_	220 to 240	0.065 nm/cycle	Thermal	Transistors, capacitors	[144]
MoS_2_	Mo(NMe_2_)_4_	N_2_	60	1.2 Å/cycle	Thermal	Catalysis, battery	[145]
CuSbS_2_	CuAMD, SbTDMA, H_2_S	N_2_	225		Low-Temperature	Photovoltaics	[146]
TiO_2_	TiCl_4_, H_2_O	N_2_	180	0.6 Å/cycle		Photocatalysis	[147]
TiO_2_	TiCl_4_, H_2_O	N_2_	40–250	0.048 nm/cycle to 0.113 nm/cycle	PEALD, tALD	Catalysis, semiconductors	[148]
V_2_O_5_ and VO_2_	VO[O(C_3_H_7_)]_3_, H_2_O	N_2_	135	0.03 nm/cycle	PEALD, tALD	Microelectronics	[149]
Ag	Ag(fod) (PEt_3_), BH_3_(NHMe_2_)		110	0.3 Å/cycle	Thermal		[150]
Bi_2_O_3_	Bi(Ph)_3_ and O_3_	N_2_	250 and 320	0.23 Å/cycle		Supercapacitors, gas sensors, solid oxide fuel cells	[151]
ZnS	ZnS/g-C_3_N_4_	N_2_	200		Thermal	Photocatalysis	[152]
Ta_2_O_5_	Ta(N*^t^*Bu)(NEt_2_)_3,_ Ta(N*^t^*Bu)(NEt_2_)_2_Cp	N_2_	250–300	0.77 and 0.67 Å/cycle	Thermal	Capacitors, solar cell	[153]
Cobalt	bis(1,4-di-*tert*-butyl-1,3-diazadienyl) cobalt and *tert*-butylamine	N_2_	170 and 200	0.98 Å/cycle	Low temperature	Microelectronics	[154]
ZnO/ZrO_2_	Tetrakis(ethylmethylamino)zirconium (TEMAZ), diethylzinc (DEZ) and H_2_O	Ar	200	1.0 Å/cycle	Thermal	Optical and electronic devices	[155,156]
HfO_2_	HfCl_4_ and H_2_O, tetrakis (dimethylamino) hafnium and H_2_O	N_2_	200–275	−0	Thermal	Transistors (MOSFET)	[157,158]
HfO_2_	Tetrakis(dimethylamino)hafnium (TDMA-Hf, [(CH_3_)_2_N]_4_Hf) and H_2_O	N_2_	250		Thermal	Microelectronics	[159]
ZrO_2_	(C_5_H_5_)Zr [N(CH_3_)_2_]_3_) and O_3_	Ar	180	0.8 Å/cycle	Thermal	Capacitors (DRAM)	[199]
ZrO2	CpZr[N(CH_3_)_2_]_3_/C_7_H_8_ and O_3_	Ar	250 to 350		Thermal	Microelectronics	[160]
ZnO	(C_2_H_5_)_2_Zn and H_2_O	Ar	100 to 300		Plasma, Thermal	Microelectronics, Solar energy	[161–163]
MoS_2_	Trimethylaluminium (TMA) and H_2_O	N_2_	200	1 Å/cycle	Thermal	Field-effect transistors (FETs)	[164]

#### 3.2.2. Reactor

Conventional ALD reactors, such as vacuum or viscous flow reactors can be used in ALD for non-planar and high surface area substrates. For the substrates with high aspect ratios, continuous flow processes sometimes require impractical lengths of exposure time for achieving full and uniform fillings of trenches because of the insufficient Knudsen flow of precursor gases. In a stop flow process, higher precursor concentration can be applied since it requires longer diffusion time to deposit on the trench surfaces. When using high-surface-area substrates, it is attractive to use dedicated reactor designs, since the use of conventional reactors will lead to longer deposition times and lower precursor efficiency [64].

Depending on the required deposited materials functionality, there are many different types of ALD reactors such as: batch reactors, compact reactors, fluidized bed reactor, and rotary reactor. The various types of typical ALD reactor systems based on gas injection are (a) Cross-flow reactor system based on forced flow laterally across the wafer, b) system with a single injector above the centre of the wafer, c) showerhead system; gas is injected through an array of injectors covering the entire wafer surface, and d) vertical batch reactor: 50–150 wafers are processed simultaneously. Table 7 gives ALD reactor types classified by their processing-related characteristics.

**Table 7.** Main ALD reactor types classified by their most important processing-related characteristics. (Adapted with permission from [165]).

Reactor	Processing ability	Gas-solid contact	Agglomeration prevention	Energy Provision	Vacuum quality
Flow-type	Medium	Flow through	None (static bed)	Thermal	Medium
Viscous flow	Low	Flow over	None (static bed)	Thermal	Medium
Fluidized bed	High	Mixed flow	Mechanical & pneumatic vibration, stirring, microjet, pulsed flow	Thermal	Medium-atmosphere
Rotary	Medium	Mixed flow	Rotational agitation	Thermal & plasma	High-medium
Pneumatic conveying	High	Local mix (jet)	None	Thermal	Atmospheric

#### 3.2.3. Effluent gas handling, emissions, energy consumption

Although ALD has great potentials, yet it has significant sustainability issues that need proper investigation and improvement prior to its full spectrum industrial applications. Among these issues, one of the key problems is the potential nano-particle emissions from the ALD fabrication process which causes occupational, public health risks and environmental impacts due to the unique properties of nanoparticles [166]. Other issues in the ALD process are that only a small percentage of the gases reacts and the greater portion is emitted into the environment [167]. For instance, in the reaction involving TMA, CH_4_ (Methane) is emitted, this is not only harmful to the environment but also has an impact on the person(s) handling the experiments. Due to the hazardous nature of TMA, alternative ALD precursors need to be investigated. For example, [3‐(dimethylamino)propyl]dimethylaluminium, [AlMe_2_(DMP)] (DMAD) [168] has been suggested as ALD precursor that can enable the development of new and promising ALD processes for Al_2_ O_3_ thin films at low temperatures and this finds more applications, nevertheless serious considerations need to be given to the emitted gases. Methane, for instance, has the main impact on a global scale as a greenhouse gas, 21 times more than CO_2_. Though its level in the environment is relatively low, it has high global warming potentials.

Yuan et al. [169] performed the sustainability analyses by investigating the film production, precursor emissions, greenhouse gas emissions, and nano-wastes generated quantitatively from the ALD Al_2_O_3_ processes. They found that huge amounts of environmental emissions are generated which implies that current ALD nanotechnologies need significant improvement before wider industrial applications.

Ma et al. [167] in the emission study of ALD found that it contains 3.22 vol% of CH_4_ and 6.01 × 10^–2^ vol% of C_2_H_6_ [167]. They also found that the net peak emissions of the aerosols were between 1 × 10^3^ and 1 × 10^4^ cm^−3^ and the net total emissions of 25 cycles were in the range of 6.0 × 10^5^ and 2.5 × 10^6^ particles [167]. In an experimental investigation of the emission process in ALD, Ma et al. [170] found that about 93% of trimethylaluminium (TMA) flowed through the chambers without deposition which implies higher emission to the environment. They also reported that 2–9 × 10^4^ of ultrafine nanoparticles containing 51.9 ± 4.6% of C, 16.6 ± 0.9% of Al, 31.4 ± 4.1% of O are generated during each cycle of reactions. They concluded that the purge time has great influence on the ALD emissions because the purge time changes the reaction as well as the degree of gas phase mixing.

**Figure 7.**
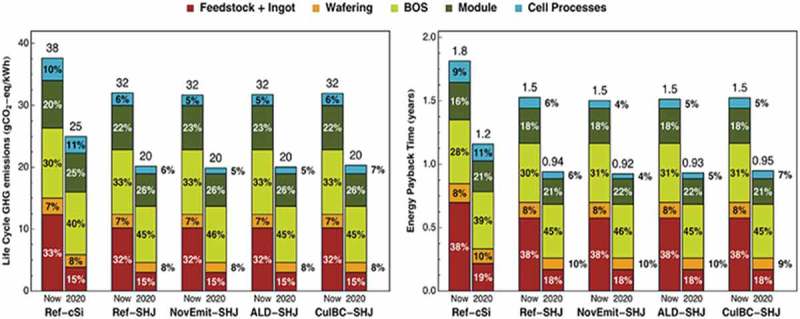
Life cycle greenhouse gas emission and cumulative energy demand in an ALD process compared to other processes. Adapted with permission from [171]. Results are modelled based on cell efficiencies of 20.4% and 25% for current and prospective cells, respectively. SHJ - Silicon heterojunction.

Louwen et al. [171] in their work on the life cycle greenhouse emission and energy payback time of current and prospective heterojunction solar cell designs; compared the ALD with other deposition techniques like the PECVD and the results are presented in Figure 7. The energy consumption of ALD equipment (expressed per m^2^ cell produced) is almost 90% lower according to the equipment data they reviewed. They concluded that ALD has a lower carbon footprint due to plasma enhanced deposition and lower cumulative energy demand. The vacuum operation that could contribute to high energy requirement and carbon footprint is also reduced for this design because the startup of the PECVD equipment (creating vacuum and preheating) is more energy intensive.

ALD design process can be significantly challenging for the chamber exhaust pumps. The chamber exhaust sends the effluent gases to the fab scrubbers. The exhaust system is responsible for making sure the two precursors only interact on the substrate surface and not on the chamber sidewalls and in the exhaust system itself. If this is not properly considered in the design phase, it could become an issue. To prevent this issue, it is necessary to increase the pump speed and separate the two precursor materials [172]. For safety purpose, pump exhaust gases must be appropriately treated before releasing them to the environment which should be properly incorporated in the design and fit for the purpose.

## 4. Applications

### 4.1. Microelectronics

#### 4.1.1. Electronics applications

As technology advances, devices were pushed towards scaled down into nano and atomic levels with more spatially distributed structures. ALD holds the potentials and advantages over other thin film deposition techniques such as CVD and PVD techniques, because of its conformality, composition and control over material thickness. Some of the characteristics of ALD that makes it a tool for nanotechnology includes: atomic scale thickness control, excellent conformality and low growth temperature [5,7]. In this work, few works on the applications in microelectronic materials have been reviewed, as follows.

#### 4.1.2. Transistors

**Figure 8.**
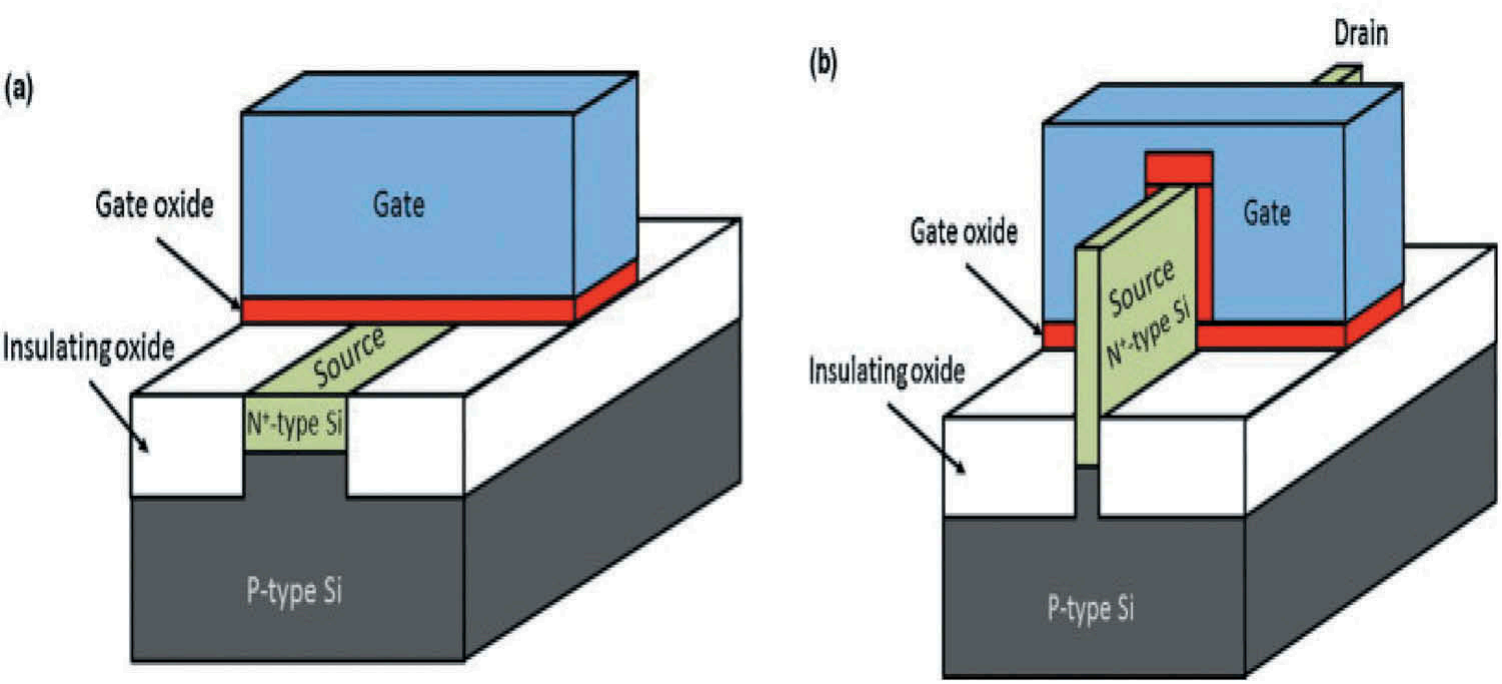
(a) The traditional planar MOSFET design leading to an inverted surface channel and (b) the FinFET or trigate design where a Si fin that is covered by the gate oxide from three sides becomes inverted from the surrounding gate oxide, thus increasing the overall inverted volume compared to the planar design for the same gate voltage. Adapted with permission from [7], copyright Sciencedirect 2014.

Developing a high ordered three-dimensional structure which provides higher surface area and consequently improving the device efficiency is a growing trend. As a result, the value of the ALD technique has been increased and the presence of ALD in manufacturing semiconductor devices will continue to increase. In the recent fabrication of transistors, the research and development are more on the use of ALD for the deposition of conformal films with well-controlled thickness, pinhole-free and high dielectric constant [7]. Several researchers have published works on the use of low-temperature ALD reactor in fabricating the transistors [173–178]. Jin et al. [179] reported the use of ALD for compositional tuning of a range of Mg_1-x_Zn_x_O to fabricate thin film transistors and also the influence of varying the composition and ratios of the oxides on the material and device characteristics. Liu et al. [180] used ALD method to deposit zinc oxide on graphene for thin film transistor. They had a high ON/OFF ratio while the thin film transistor of the zinc oxide on graphene showed enhanced carrier mobility over zinc oxide thin film transistor. In the fabrication of high performance p-type thin film transistors using ALD on silicon oxide films, Kim et al. [181] operated the reactor temperature at 210 °C to suppress the hole carrier concentration effectively. This gave a high ON/OFF ratio and high field-effect mobility though defects and hole carriers. The post-deposition process back channel surface passivation with ALD grown Al_2_O_3_ at 250 °C post-annealing reduces the defects considerably leading to a superior thin film transistor performance. Figure 8 gives an illustration of ALD application in a transistor. Figure 8(a) shows a traditional planar metal-oxide-semiconductor-field-effect transistor (MOSFET) design while Figure 8(b) shows Fin field effect transistor (FinFET) trigate design which has been enhanced using the process of ALD. The Si fin which is covered by the gate oxide on three sides is inverted therefore increasing the overall surface area compared to the traditional MOSFET design as shown in Figure 8(a).

**Figure 9.**
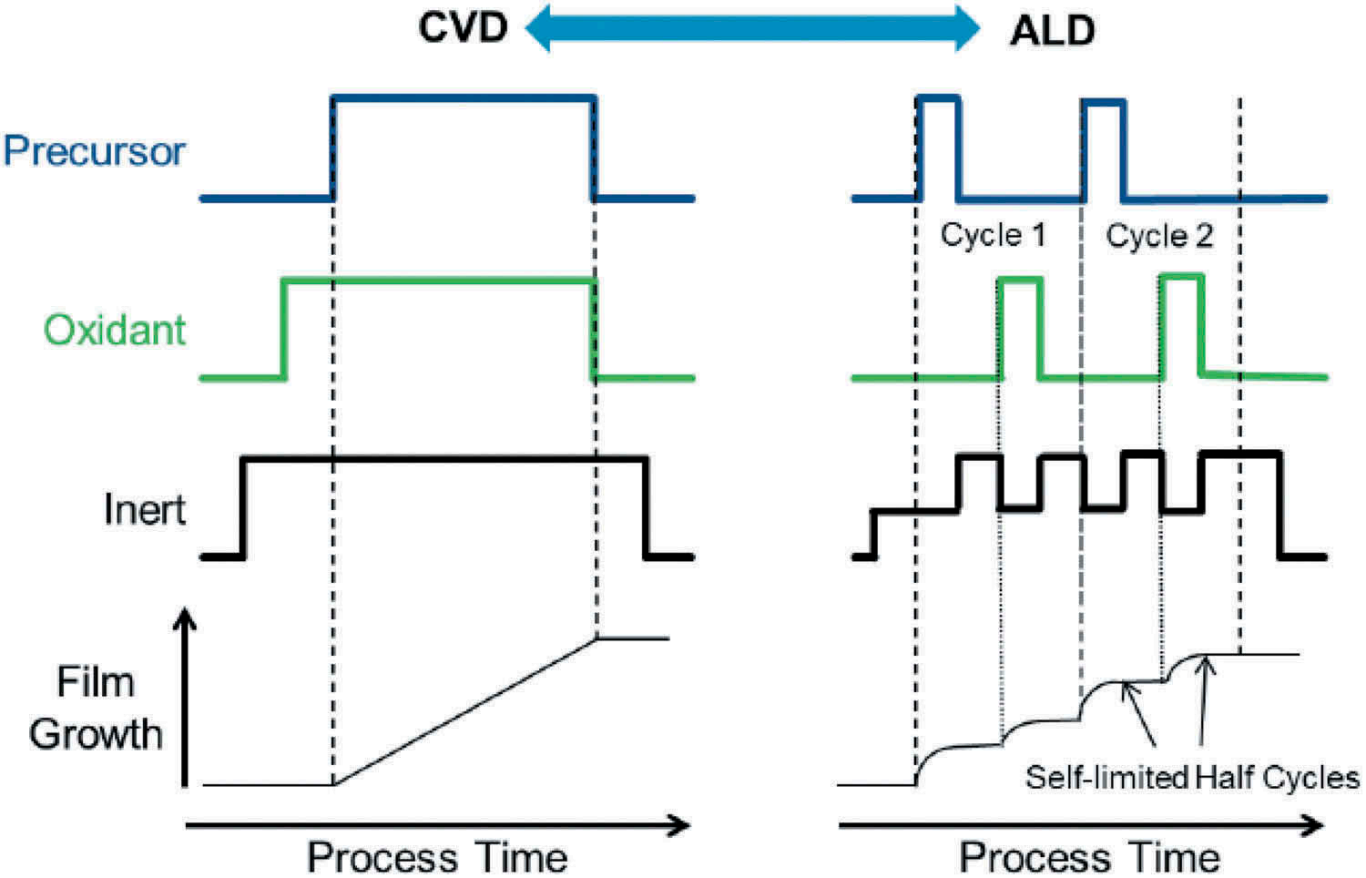
Schematic showing a basic gas flow sequence for Chemical Vapour Deposition (CVD) and for Atomic Layer Deposition (ALD) as well as expected film growth profiles vs. process time. Adapted from [194].

In microelectronic industry emerging applications for microelectronic and semiconductor industry in very large-scale integrated circuit technology are being developed. Figure 9 shows the CVD and ALD process with respect to gas flow sequence. The flow sequence and the expected film growth profiles vs. process time are shown in Figure 9. The deposition rate is continuous for the CVD while the ALD is a cyclical deposition in which the half cycle is self-limited due to the surface chemistry.

More information on the influence of the composition of materials on devices and the effects of temperature transistors through the application of the ALD process can be found in the papers referenced [182–193].

#### 4.1.3. Capacitors

Atomic layer deposition on porous alumina membranes promises a path to produce high-performance capacitors [195]. Groenland et al. [196] studied metal insulator silicon (MIS) and metal insulator metal (MIM) capacitors using titanium nitride. They applied the atomic layer deposition process in the integration of conductors on insulation materials. Further research was done by Dustin et al. [197] used the PEALD to develop Al_2_O_3_/SiO_2_ based metal-insulator-metal-capacitors. They employed the cancelling effects between positive quadratic voltage coefficients of capacitance (αVCC) of aluminium oxide Al_2_O_3_ and the negative quadratic voltage coefficients of capacitance (αVCC) of silicon oxide (SiO_2_). With mainstream materials and low processing temperature, they achieved better leakage current density, capacitance density and voltage nonlinearity [197,198]. More researches have been published on metal-insulator-metal capacitors using ALD [198–201]. More work on the application of ALD for capacitors has been done by different researchers looking at the growth rate, limitations and various applications on different materials [202–207].

**Figure 10.**
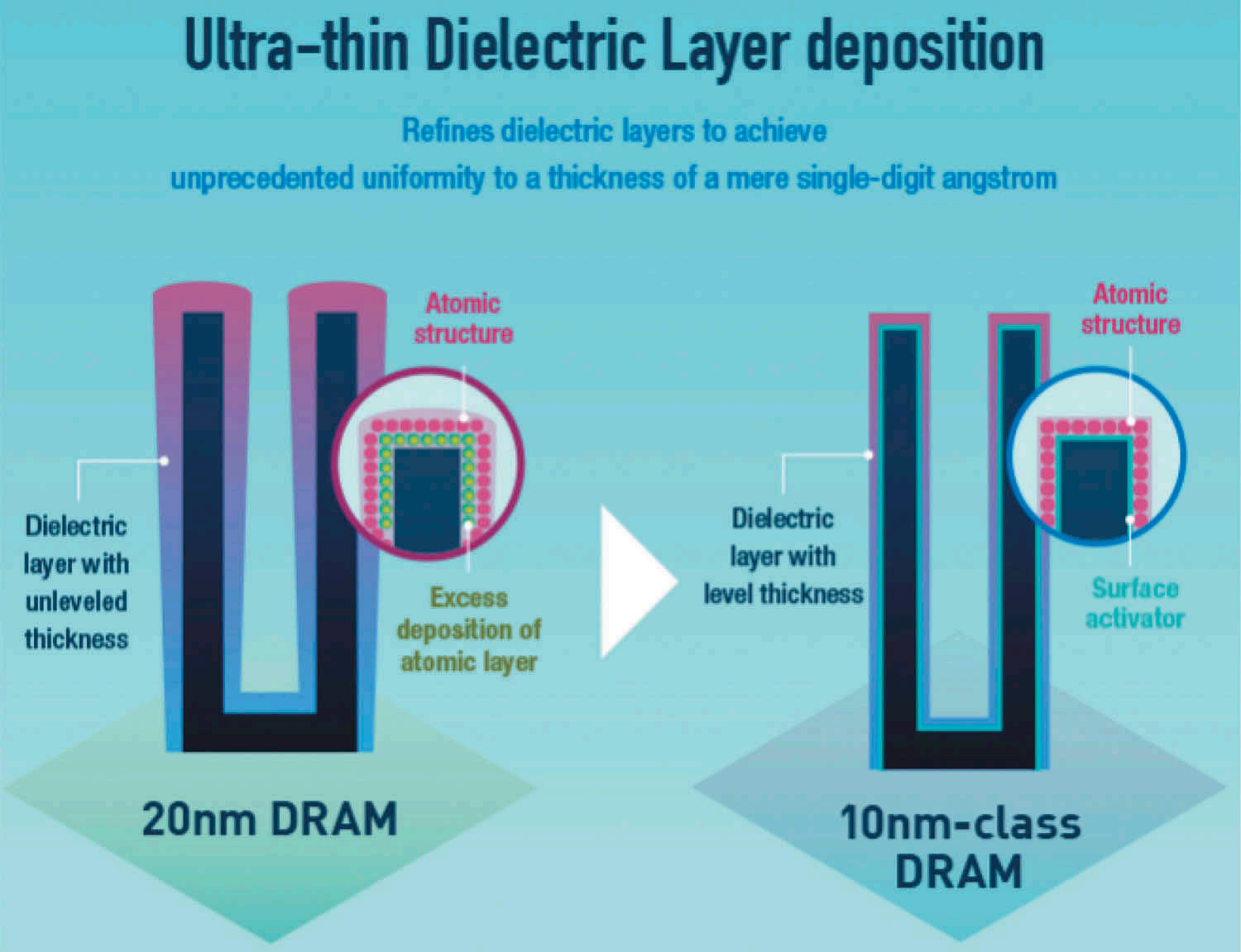
The new 10 nm-class DRAM with high performance and reliability by Samsung. The thickness of the dielectric layers uniform to a few angstroms-DRAM chip contains hundreds of millions to billions of cells depending on data capacity. Each cell consists of two parts: a capacitor that stores data in the form of an electrical charge, and a transistor that controls access to it. Adapted from [208].

Figure 10 illustrates the new 10-nm-class Dynamic random-access memory (DRAM) with high performance and reliability developed by Samsung. The DRAM which is an 8 Gb (gigabit) chip has more than 8 billion cells. Each of the cells in the chips consists of a capacitor that stores data in the form of electrical charge and the transistor that controls access to it. ALD was applied to achieve this feat that enhances system performance.

### 4.2. Energy storage and conversion

Fuel cell technology is a promising and reliable clean energy technology due to their low emission of pollutants and high energy conversion efficiency [23]. Among the fuel cell types are polymer electrolyte membrane fuel cells (PEMFCs), direct methanol fuel cell (DMFCs), molten carbonate fuel cells (MCFCs), solid oxide fuel cells (SOFCs), reversible fuel cell, phosphoric acid fuel cells (PAFCs) and alkaline fuel cells (AFCs). Introduction of ALD to this field could significantly lower the costs of fuel cells and increase the life-span of the fuel cells. Catalysts as a part of fuel cells are a major contribution to both the high cost and limited durability of fuel cells [23]. In catalysis, the larger surface-area-to-volume ratio is required therefore particles has preference compared to films. As a common example is to improve the catalytic activity and performance of Pt electrode in fuel cells which can be achieved by modifying the catalytic layer. ALD could be introduced to effectively address the earlier challenges encountered such as high Pt loading, conformity and durability and also could improve the design of the catalysts [209].

**Figure 11.**
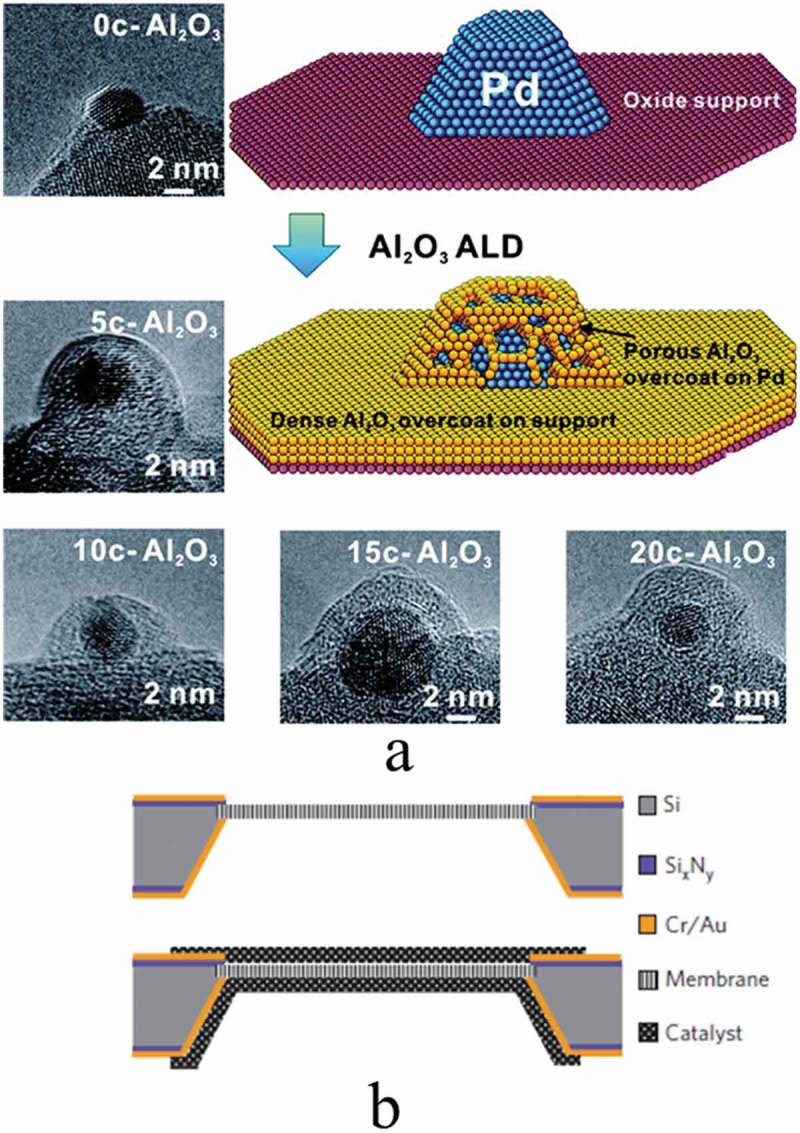
(a) TEM images of spherical alumina supported Pd catalysts with different numbers of Al_2_O_3_ ALD overcoating from 0 to 20 cycles and schematic illustration of porous ALD Al_2_O_3_ overcoat on Pd NP for Oxide-supported Pd catalyst and dense Al_2_O_3_ film on oxide support and porous Al_2_O_3_ overcoat on Pd NP formed by ALD. Republished with permission from [211], copyright 2012 American Chemical Society, b) Cross-sectional schematic of a single membrane within its silicon die before and after application of the catalyst layers using ALD. Adapted with permission from [210], copyright Nature 2010.

Lu et al. [211] reported in porous alumina protective coatings of palladium from poisoning by applying atomic layer deposition as shown in Figure 11(a). They sought to understand how the catalytic activity of the Pd nanoparticles was maintained after ALD of Al_2_O_3_ cycles expected to bury the Pd nanoparticle surface and concluded that conformal Al_2_O_3_ films on the Pd nanoparticle surface were achieved.

Jiang et al. [212] created a thin hydrophilic silica aperture at the mouth of the pores, using plasma-directed atomic layer deposition (PD – ALD). Figure 11(b) illustrates a cross-sectional schematic of a single membrane before and after the application of catalyst on its surface. The chromium/gold layers already deposited on both sides of the die are used as current collectors. The catalyst layer overlaps with the chromium/gold electrode around the edges of the membrane and provides electrical connectivity. The ability to modify the surface of stable membrane enabling the development of better fuel cells.

### 4.3. Desalination

Freshwater can be obtained from the ocean, sea and brackish waterbodies through the desalination process. However, this process can be costly. In another hand, seawater accounts for about 97.5% of the water resources on earth [213]. Desalination has become increasingly significant for water production in semi-arid coastal areas [214,215]. It is no gainsaying that the world’s growing population would need this water source to meet its growing demands. Several technologies are being used in the desalination process, the most desalination technologies at present are based on membrane separation through the reverse osmosis (RO) and thermal distillation (multistage flash (MSF) and multi-effect distillation (MED)) [216]. Reverse osmosis based on polymeric membranes has several challenges which include slow water transport and tremendous energy costs [109,217,218]. Hence, the need for a method to resolve this issue and biomimetic membranes. An emerging technology has promising potentials to address the issues. This review paper will focus on the latest development of biomimetic based membranes.

#### 4.3.1. Biomimetic membrane

Learning from nature has become a common philosophy among the technical and scientific communities. Biomimetic means the study of structures and the functioning of biological systems and developing the model for the design of engineering solutions [219]. Bio-inspiration and biomimetic are now used in many fields such as chemical, materials science, pharmaceuticals, the medical fields, carbon capture and clean energy [220,221]. Different methods have been used in functionalizing a variety of materials for this purpose [222]. According to Zhao et al. [220], ideally biomimetic and bioinspired membranes should possess the following features:

- Fabrication of the membrane through self-assembly under conditions close to the natural atmosphere such as atmospheric pressure, room temperature and aqueous environment.
- The membranes are materials with excellent hydrodynamic, mechanical, wetting and adhesive properties usually composed of the lightest elements in the first two rows of the periodic table.
- The hierarchical organization of the membrane structure spans from molecular-nanoscale-microscale-macroscale bearing controlled configuration, mutable surface and robust interface.
- The membrane properties are highly dependent on the content and state of water in the structure, and membrane processes can be intensified by rationally manipulating the multiple selectivity mechanisms.

Currently, researches are ongoing on ways to integrate high performance bioinspired and biomimetic membranes in industrial and municipal water treatment, but membrane fouling is of great concern in the implementation of membrane water treatment because it increases cleaning requirements, energy consumption and drastic flux decline. The biomimetic membrane can introduce better strategies in developing a range of antifouling membranes which have potentials for better separating capability [221].

Aquaporin which has high water permeability and high solute rejection have attracted interest as functional building blocks for biomimetic membranes in water desalination and water reuse [36].

Susan and team in the Sandia laboratory patented a filter designed mimicking nature (Biomimetic) with porous membranes with controlled nanopores architecture and controlled chemistry through ALD [37,38]. In their work, they developed a biomimetic membrane for water desalination by using ALD technology. The ALD based biomimetic membranes which are centred on selective water transport and solute rejection demonstrate higher water flux and more efficient than commercial membranes available.

**Figure 12.**
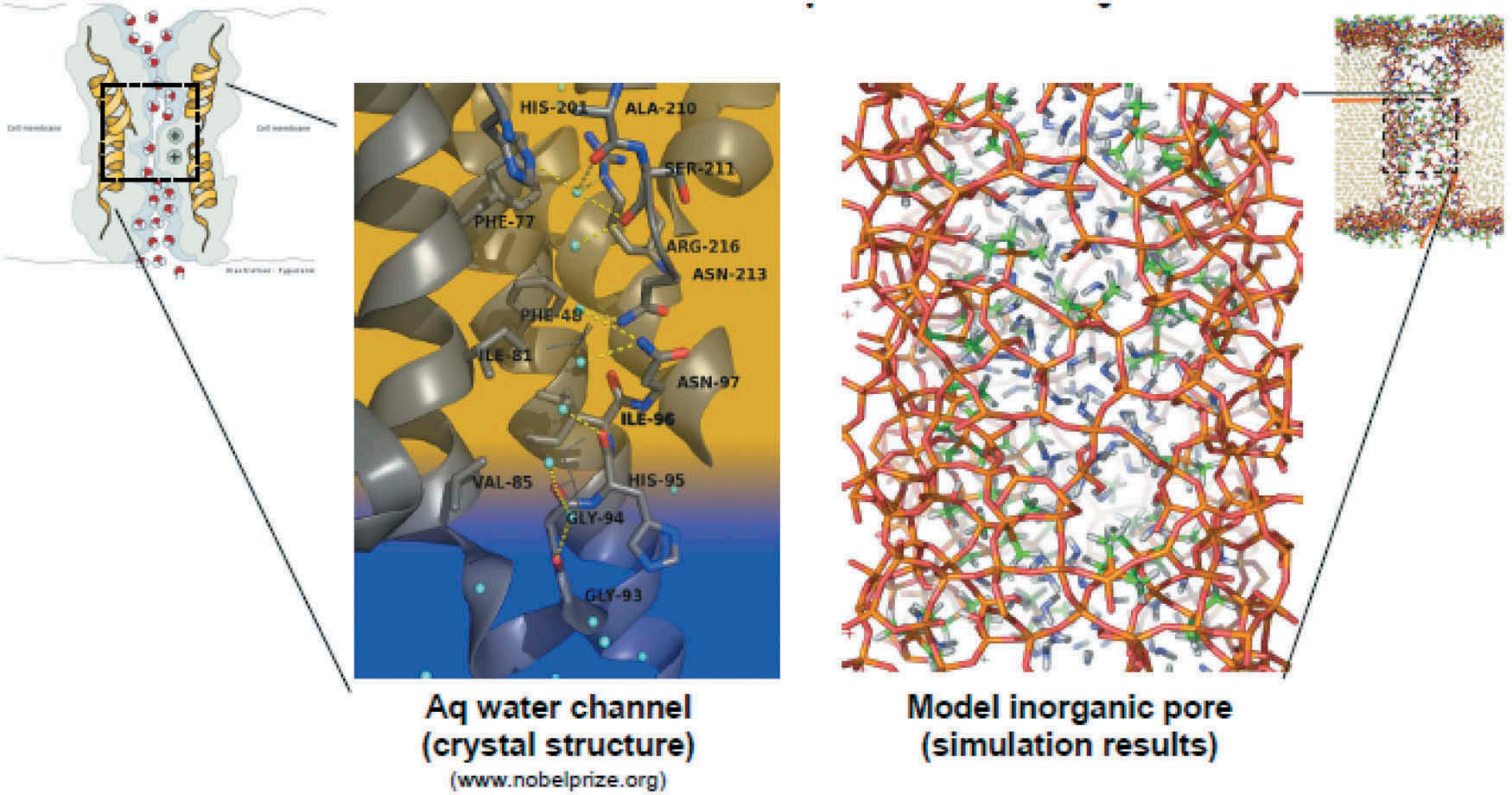
Complex structural features of biological pores that can be adapted for biomimetic filtration. Adapted from [38].

The biological aquaporin protein channel – a cellular membrane found in the kidney and other cells. with water transport rates of 10^9^ per pore per second with the complete rejection of ions are shown in Figure 12 (left). This provides a working example of a membrane pore that demonstrates fast water flux and selective ion rejection through nanopores at low applied pressure than current reverse osmosis membranes. It shows the complex structural features of the biological pores, short narrow passageway for water and the repulsive hydrophobic walls and the staircase of stabilising polar groups.

Figure 12 further shows a series of *hydrophilic* carbonyl oxygens and amine groups that form a staircase along the other side which is part of the biological filtering mechanism and model for the selective desalination process. Representative snapshot of the silica pore simulated is shown in the right diagram of Figure 12. The Silicon (Si) atoms are coloured orange, the oxygen (O) atoms of silica are coloured red, the Carbon (C) atoms of the methyl groups are coloured green, the Oxygen (O) atoms of the water molecules are coloured blue, and all Hydrogen (H) atoms are coloured white. The simulated system was studied, and the membrane synthesized applying the several methods such as self-induced evaporation and ALD to achieve a membrane with high permeation and better salt rejection.

With the system (desalination using ALD tuned membranes), lower energy cost reduction of about 88% and high filtration can be achieved [38]. Two important factors that define the effectiveness and performance of a water desalination membrane are ion rejection and water flux which can be addressed effectively through the ALD process.

**Figure 13.**
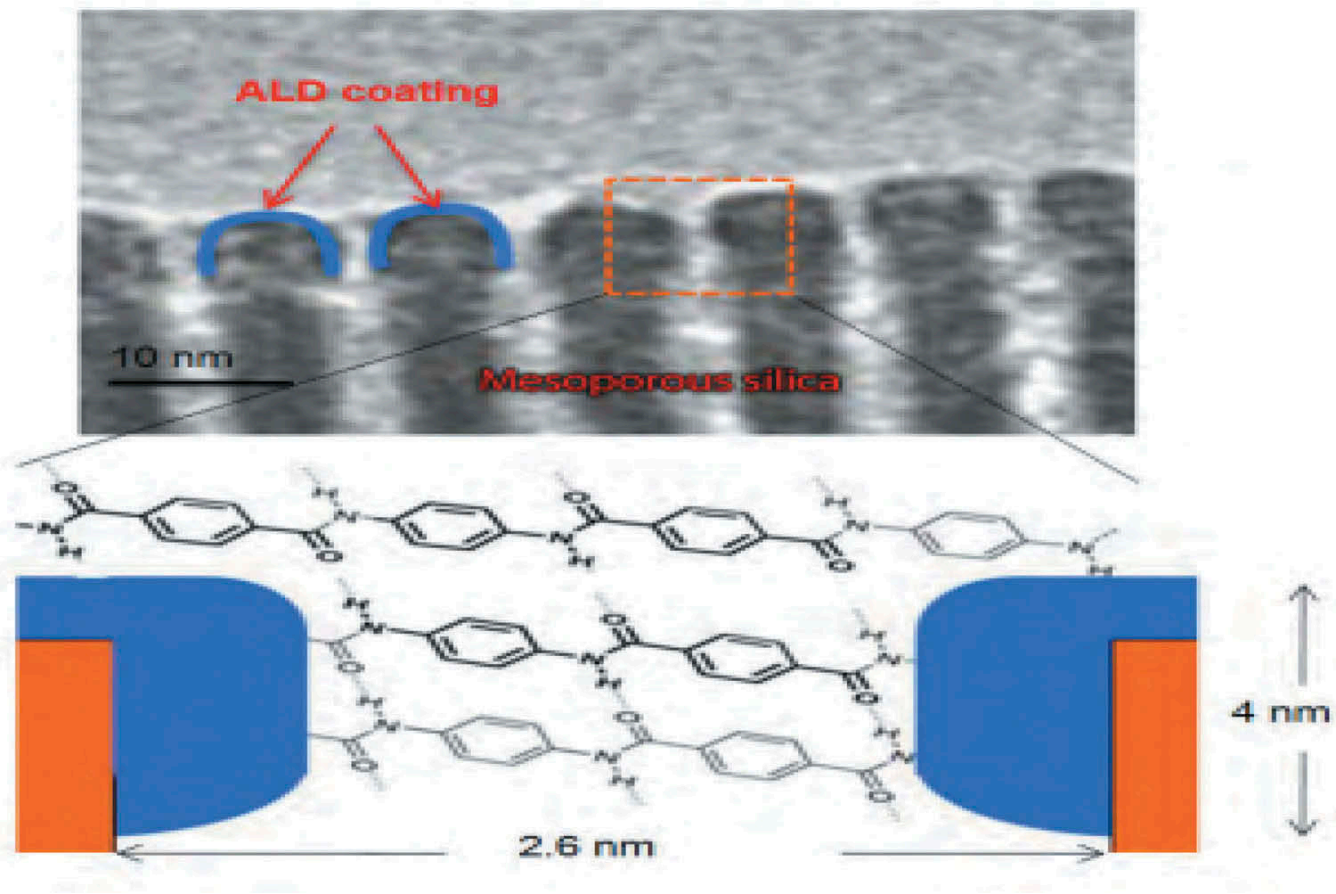
Translated biomimetic design transmission electron microscopy (TEM) showing pore geometry modifications achieved by atomic layer deposition targeted to the pore mouth. ALD of polypeptide groups which modifies internal pore chemistry to produce pore active sites with dimensions and chemical functionality similar to natural biological pores. Adapted from [38]. (Pictures taken from Sandia laboratory publication Biomimetic membrane for water purification 2010 [38]).

ALD, a self-limiting step by step ultra-thin deposition process, was applied in the synthetic nanoporous biomimetic membrane as shown in Figure 13. The channel which is 2.6 nm diameter was functionalised using polypeptides resulting in *hydrophobic* and *hydrophilic* functionality in the pore active through the plasma enhanced ALD process. As a result, a small narrow constriction is formed near the surface which rejects Na^+^ and Cl^−^ but allows water to pass through.

Membranes which could include nanoporous materials can be formed through an evaporation-induced self-assembly process forming uniform pore sizes [223,224]. The porous materials could be further tuned to mimic the architecture and chemistry of the biological system hence attain the desired chemical and selective chemical giving the required flux that the natural biological system possesses. Figure 13 shows the translated biomimetic design transmission electron microscopy (TEM) showing pore geometry modifications achieved by atomic layer deposition targeted to the pore mouth. The support was formed from anodized alumina in this instance but could also be polymers (polysulfone, polyamide, and polycarbonate), ceramics, cermets, metals, alloys, glass and carbon. For water permeation, the pore radius should be equal to or greater than about 0.14 nm after functionalization [38,223]. The functionalised surface could be of amines, carboxylates, nitrides and nitriles; in Figure 13 the as bridged material achieved through ALD, is silsesquioxanes (RO)_3_S – R′ – Si(OR)_3_ where R is typically an alkyl group and R′ and organic ligand including methane (CH_2_), ethane (C_2_H_4_), phenylene, resulting in a hybrid organic-inorganic material.

Desalination with aquaporin-based bio-inspired membranes holds great potentials compared to current water filters; however, it still has not been fully developed for industrial application partly due to the stability of the membranes during real operations. Only a few studies [37,38,225–228] have been done on biomimetic membranes. However, despite the difficulties, this type of membrane holds great potentials in more productive and sustainable water treatment for solving the water crises in the area where they have saline water. Substantial progress in the permeability of solute-rejecting membranes would mean a great step in improving the economics of desalination for drinking water applications. There are open doors for further research to develop the biomimetic membranes on alternative flexible supports to enable scale-up with temperature, fouling chemical attacks, mechanical stability and pressure effects in perspective which ALD can be an alternative to overcome the constraint of membrane film development.

#### 4.3.2. Graphene

**Figure 14.**
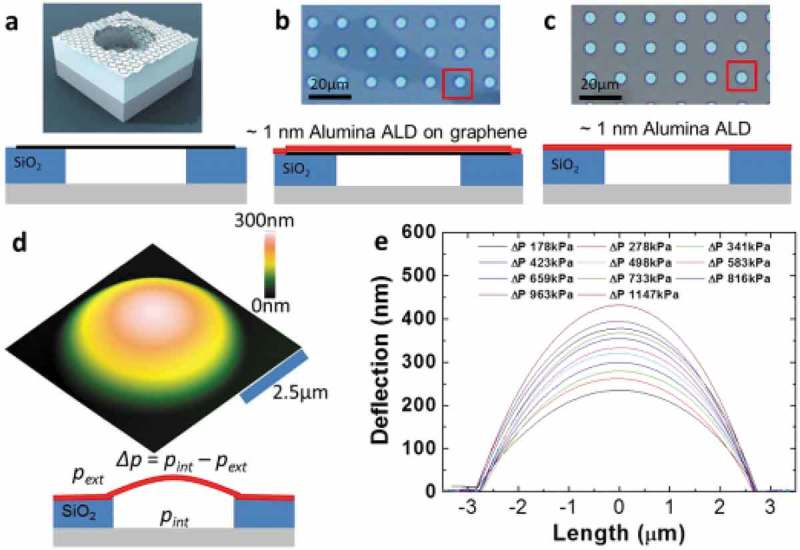
(a) Schematic of a graphene membrane before atomic layer deposition (ALD). (b) Optical image of an exfoliated graphene flake with 7 cycles of alumina ALD. (c) Optical image of a pure alumina film after graphene is etched away. (d) Atomic force microscope image of a pressurized 7-cycle pure alumina ALD film with ∆p = 278 kPa. (e) Deflection vs. position through the centre of the film in (d) at different ∆p. Adapted with permission from [232], copyright Nanoletters 2012.

Graphene which is a two-dimensional material with extraordinary properties finds application for many optical and electronic devices and a future for nanoelectronics and many nanotechnology applications. In addition, it has mechanical properties which are good for nanomechanical systems, transparent and conductive composites and thin film transistors [229]. Graphene has high charge carrier, good thermal conductivity, large maximum current density and good light absorbance over broad spectra [230]. Significant development has been made in preparation of large-area graphene. Graphene with very high intrinsic charge carrier mobility (more than 200,000 cm^2^/Vs at 4.2 K) and thermal conductivity above 2000 W/(m.K) at room temperature has become the promising material for electronic and optical applications [230,231]. Integration of graphene in microelectronics devices requires the deposition of thin dielectric layers on top of graphene which brings the challenge. Thanks to the good deposited ultrathin layers of ALD, which generate high-quality films with sub‐monolayer thickness control [230]. Wang et al. [232] created an ultrathin film based on aluminium oxide of ALD on graphene. The film which is mechanically robust, pinhole free and thickness in nanometre, and has Young’s modulus of 154 ± 13 GPa. These films can be integrated with graphene or other nanomechanical structures to create multifunctional electromechanical structures. They find applications in fields such as thin film coatings, membranes and flexible electronics. The schematic of the graphene membrane before ALD and the optical image after cycles of ALD with the atomic force microscope images is shown in Figure 14.

According to Alles et al. [233], for the ALD of high-quality dielectric layers, a seed layer is grown using highly reactive precursors and low deposition temperature or through functionalization of the graphene surface with a metal or polymer buffer layer. However, this could lead to degradation of the electronic properties of graphene or result in a higher high-k dielectric layer. It has been suggested that further experiments are required for the optimization of the graphene parameters to derive its full electronic potential.

**Figure 15.**
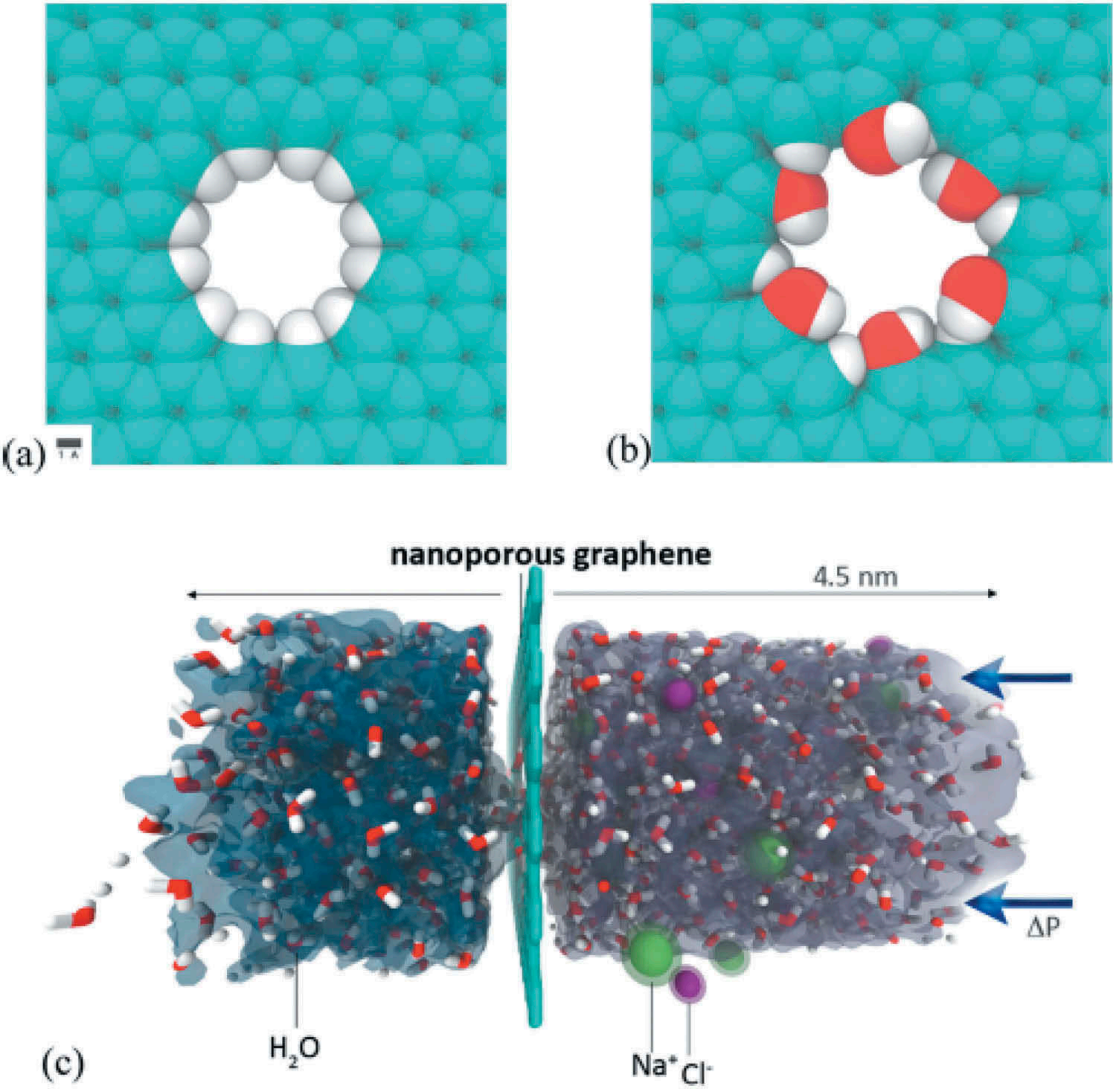
(a) Hydrogenated graphene pore (b) and hydroxylated graphene pore, and (c) side view of the computational system. Adapted with permission from [228], copyright Nanoletters 2012.

Owing to the exceptional properties of graphene, it finds many applications and has the potential to address the issues of water desalination and climate change. Cohen-Tanugi et al. [96,234,235] demonstrated this through their simulation work on desalination using graphene membranes as a function of pore size, chemical functionalization and applied pressure to effectively address the high energy required in the reverse osmosis system. The pore size and surface chemistry functionalized through hydrogenation and hydroxylation are shown in Figure 15. The fabrication can be effectively done through the ALD process. In mimicking nature for the water transport, graphene oxide membrane was functionalized with aquaporin-mimicking peptides [236]. Even though it is not through the ALD process, a similar result was achieved compared to the result from Sandia laboratory which is through the process of ALD [38].

### 4.4. Catalyst

Catalysis is an essential technology for accelerating and directing chemical transformations. ALD is a powerful tool for atomically precise design and synthesis of catalytic materials. It is key to develop new technologies in creating values from sources such as fossil fuel, biomass, carbon dioxide and water. It finds application in major industries such as energy, pharmaceuticals, healthcare, chemicals, agriculture, food processing, consumer products and environmental remediation [237]. The grand challenge is how to improve the selectivity of catalysts to convert the specific feed to specific products with little or no waste in conjunction with undesirable reactions.

**Figure 16.**
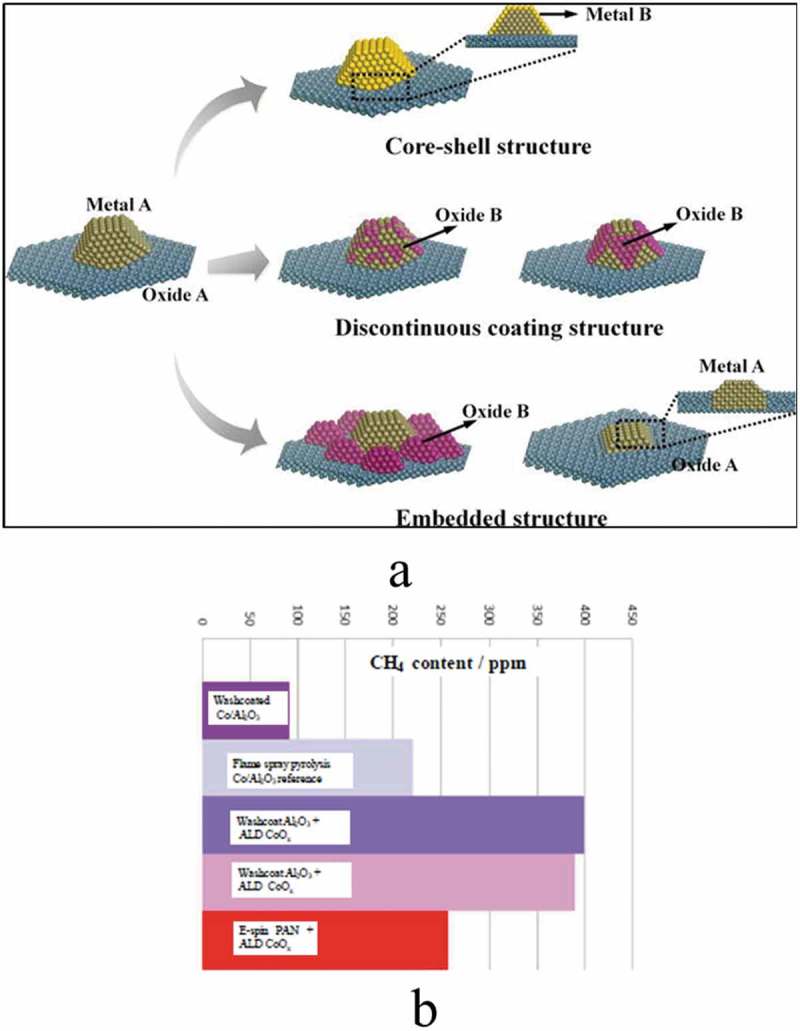
(a) Classification of composite catalysts synthesized with selective ALD in this review, (I) core-shell structure, (II) discontinuous coating structure, and (III) embedded structure. Republished with permission from [238], copyright 2017 Author(s). (b) The effect of different catalysts on Fischer-Tropsch synthesis CH_4_ output measured by Gas Chromatograph (Adapted with permission from [239]).

The task of simultaneously improving catalytic activity, selectivity and stability are common for both organic and inorganic catalysts, and a central goal is to control the size, shape, and morphology of supported nanoparticles to improve selectivity. Tailoring catalysts with atomic-level control over active sites and composite structures are of great importance for advanced catalysis [238]. Figure 16(a) shows the preparation of core-shell nanoparticles with three strategies and their applications through ALD include the core-shell structures, discontinuous coating structures, and embedded structures. ALD has been shown to be effective at controlling metal and metal oxide active sites and improving catalytic activity, selectivity, and longevity. More importantly, ALD is an effective method in making uniformly dispersed catalyst on large surface area supports. Putkonen et al. [239] studied the effect of ALD on the improvement of other methods to fabricate good performance catalyst in the Fischer-Tropsch process. They showed that combining ALD with washcoating method for catalyst preparation resulted in a highly active catalyst. This comparison is illustrated in Figure 16(b). The Fischer-Tropsch process uses a collection of chemical reactions to convert mixtures of hydrogen and carbon monoxide into liquid hydrocarbons. The Fischer-Tropsch process is now a method of choice for the synthesis of petroleum substitutes. The highest activity was obtained with metal plates having Al_2_O_3_ by the washcoating method and CoO_x_ by ALD which clearly shows the positive influence of ALD.

They also prove that ALD overcoating could suppress the commercial catalysts deactivation which happens during steam reforming, especially by thin Al_2_O_3_ layers.

**Figure 17.**
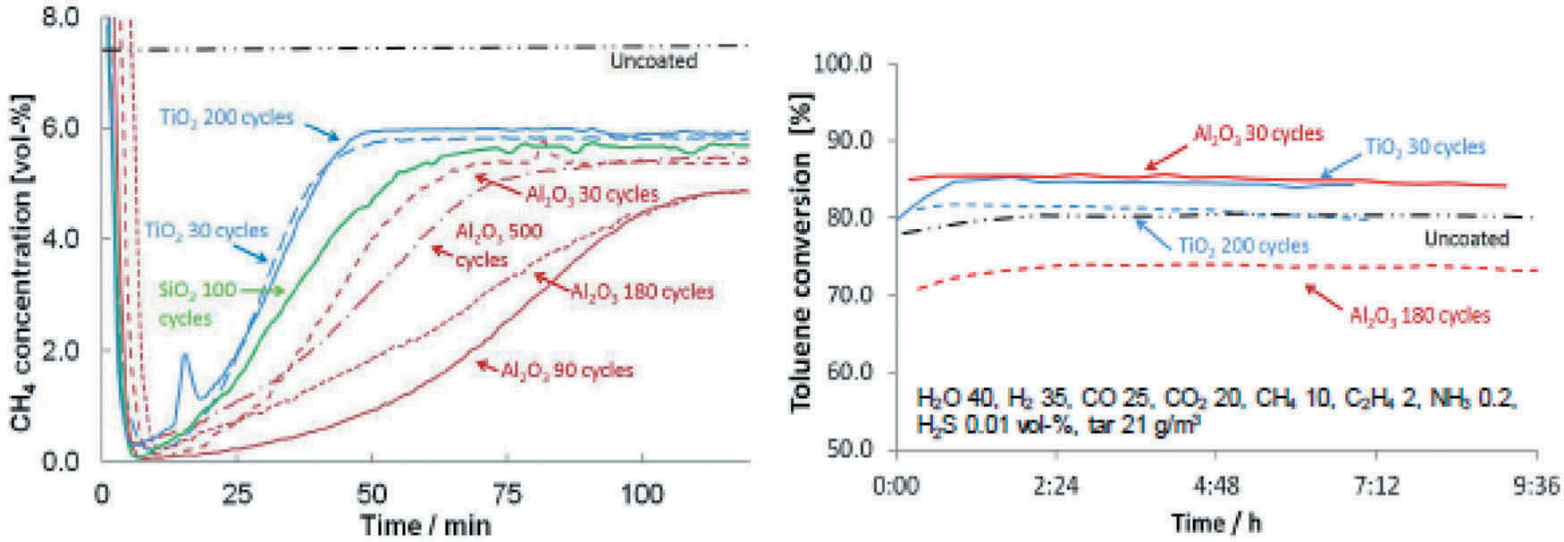
The effect of ALD oxide layers on the commercial nickel-based catalyst (left) and on commercial noble metal catalyst (right) activity on steam reforming (Adapted with permission from [239]).

In an attempt to synthesize some organic catalysts, the effect of the application of ALD for catalyst preparation was evaluated. In this study, the efficiency of CH_4_ and toluene conversion was studied using thin film catalyst and the effect of the number of film layers created by ALD was compared. As it can be seen from Figure 17, there was a difference in the product conversion for methane, ethylene and toluene with noble metal catalysts where ALD was used to prepare the catalyst with different thicknesses. Thinnest ALD coating had the highest improvements which show the ability of ALD in fabricating thin film.

To further demonstrate the growing application of ALD, a group of scientists developed a testing tool – integrated atomic layer deposition synthesis-catalysis (I-ALD-CAT) for catalyst synthesis and performance evaluation of a catalyst. It combines an ALD manifold with a plug flow reactor system. The I-ALD-CAT was successfully used in synthesizing platinum active sites, Al_2_O_3_ overcoats and in the activity evaluation of propylene hydrogenation under plug-flow conditions [240].

### 4.5. Medical

Numerous emerging flexible sensing technologies which can be used for many physical and physiological measurements in the medical fields are available [241]. Since the demand for these applications is increasing so also the processes to get effective ways of implementing them. ALD finds applications in developing some of these materials and devices.

Practically ALD using low deposition temperature has already been used in the manufacturing of flexible organic field-effect transistors (OFETs) [242]. The OFETs find application in transducing mechanical and chemical stimuli into electrical signals [243].

ALD is also finding applications in the fields of plasmonics, nanoscience and nanobiotechnology. The coatings can be used to modify the metallic surfaces and tune the optical and plasmonic properties which protect the surface from oxidation and contamination to create the required biocompatible surface [244].

ALD could also find application in nanostructured materials which are useful for the delivery of a pharmacological agent at a precise rate and specific locations in the body [245]. These nanostructured materials have applications in orthopaedic implants or self-sterilizing medical devices. In a study, Bilo et al. [246] demonstrated that ALD is a suitable coating technique to prevent metal diffusion from medical implants.

ALD has also been used in the design of nanoparticles, and larger-sized, single drug powder particle applications, as well as on tablet formulations [247]. ALD applications in active pharmaceutical ingredients are also being developed (APIs) [248].

## 5. Conclusions

This research review highlights the importance of atomic layer deposition (ALD) for different applications. ALD is becoming one of the most promising methods for depositing and producing high-quality thin film. It has proven and shown its potential to meet the demand for a variety of applications. Emerging branches of the technology are setting new opportunities and challenges for ALD processes to meet this continuous expansion of the material selection and divergent needs for conformal, high quality, and ultra-thin films. This study has gathered the information on ALD techniques in both aspects, experimentally and computational, and has compared their pros and cons with other methods. Some of the main drawbacks are the precursor wastage, energy wastage, time required for chemical reactions, nano-particles emissions and the economic viability. The advantages include the film conformity, deposition on challenging substrates, stoichiometric control and the inherent film quality associated with self-limiting, self-assembled nature of the ALD mechanism.

It has also summarized different applications in the selected fields of ALD up-to-date. Finally, this can give good and quick guidance to those researchers and practising engineers who wish to use ALD as the tool form research and engineering applications.
